# Supplementary material for: Comprehensive review of statistical methods for analysing patient-reported outcomes (PROs) used as primary outcomes in randomised controlled trials (RCTs) published by the UK’s Health Technology Assessment (HTA) journal (1997–2020)
Source: BMJ Open. 2021 Sep 6;11(9):e051673. doi: 10.1136/bmjopen-2021-051673 (PMC8422492; doi:10.1136/bmjopen-2021-051673)
Supplement: Author's manuscript [file bmjopen-2021-051673.draft_revisions.pdf]

BMJ Open

BMJ Open is committed to open peer review. As part of this commitment we make the peer review history of every article we publish publicly available.

When an article is published we post the peer reviewers' comments and the authors' responses online. We also post the versions of the paper that were used during peer review. These are the versions that the peer review comments apply to.

The versions of the paper that follow are the versions that were submitted during the peer review process. They are not the versions of record or the final published versions. They should not be cited or distributed as the published version of this manuscript.

BMJ Open is an open access journal and the full, final, typeset and author-corrected version of record of the manuscript is available on our site with no access controls, subscription charges or pay-per-view fees (http://bmjopen.bmj.com).

If you have any questions on BMJ Open's open peer review process please email info.bmjopen@bmj.com

BMJ Open

A comprehensive review of statistical methods for analysing patient-reported outcomes (PROs) used as primary outcomes in randomised controlled trials (RCTs) published by the United Kingdom's Health Technology Assessment (HTA) Journal (1997-2020)

Journal:	*BMJ Open*
Manuscript ID	bmjopen-2021-051673
Article Type:	Original research
Date Submitted by the Author:	26-Mar-2021

Complete List of Authors:	Qian, Yirui; The University of Sheffield School of Health and Related Research,<br>Walters, Stephen; The University of Sheffield School of Health and Related Research<br>Jacques, Richard; The University of Sheffield School of Health and Related Research<br>Flight, Laura ; The University of Sheffield School of Health and Related Research

Keywords:	STATISTICS & RESEARCH METHODS, AUDIT, PUBLIC HEALTH

SCHOLARONE™
Manuscripts

BMJ

I, the Submitting Author has the right to grant and does grant on behalf of all authors of the Work (as defined in the below author licence), an exclusive licence and/or a non-exclusive licence for contributions from authors who are: i) UK Crown employees; ii) where BMJ has agreed a CC-BY licence shall apply, and/or iii) in accordance with the terms applicable for US Federal Government officers or employees acting as part of their official duties; on a worldwide, perpetual, irrevocable, royalty-free basis to BMJ Publishing Group Ltd ("BMJ") its licensees and where the relevant Journal is co-owned by BMJ to the co-owners of the Journal, to publish the Work in this journal and any other BMJ products and to exploit all rights, as set out in our licence.

The Submitting Author accepts and understands that any supply made under these terms is made by BMJ to the Submitting Author unless you are acting as an employee on behalf of your employer or a postgraduate student of an affiliated institution which is paying any applicable article publishing charge ("APC") for Open Access articles. Where the Submitting Author wishes to make the Work available on an Open Access basis (and intends to pay the relevant APC), the terms of reuse of such Open Access shall be governed by a Creative Commons licence – details of these licences and which Creative Commons licence will apply to this Work are set out in our licence referred to above.

Other than as permitted in any relevant BMJ Author's Self Archiving Policies, I confirm this Work has not been accepted for publication elsewhere, is not being considered for publication elsewhere and does not duplicate material already published. I confirm all authors consent to publication of this Work and authorise the granting of this licence.

For peer review only

A comprehensive review of statistical methods for analysing patient-reported outcomes (PROs) used as primary outcomes in randomised controlled trials (RCTs) published by the United Kingdom's Health Technology Assessment (HTA) Journal (1997-2020)

Yirui Qian[1], Stephen Walters[1], Richard Jacques[1], Laura Flight[1]

[1]School of Health and Related Research, University of Sheffield, Sheffield, UK

Corresponding author:

Professor Stephen Walters

Email: s.j.walters@sheffield.ac.uk

School of Health and Related Research
University of Sheffield
30 Regent St
Sheffield
S1 4DA

Word count: 3694 (excluding titles pages, abstract, references, figures and tables)

Keywords: STATISTICS & RESEARCH METHODS, AUDIT, PUBLIC HEALTH.

For peer review only

Abstract (max. 300 words)

Objectives: To identify how frequently patient-reported outcomes (PROs) are used as primary and/or secondary outcomes in RCTs and to summarise what statistical methods are used for the analysis of PROs.

Design: Comprehensive review.

Setting: RCTs funded and published by the United Kingdom's (UK) National Institute for Health Research (NIHR) Health Technology Assessment (HTA) Programme.

Data sources and eligibility: HTA reports of RCTs published between January 1997 and December 2020 were reviewed.

Data extraction: Information relating to PROs use and analysis methods was extracted.

Primary and secondary measures: The frequency of using PROs as primary and/or secondary outcomes; statistical methods that were used for the analysis of PROs as primary outcomes.

Results: In this review, 37.9% (114/303) of trials used PROs as primary outcomes, and 83.4% (251/303) of trials used PROs as secondary outcomes from 303 NIHR HTA reports of RCTs. In the 114 RCTs where the PRO was the primary outcome, the most used PRO was Short-Form 36 (8/114), the most popular methods for multivariable analysis were linear mixed model (45/114), linear regression (29/114) and analysis of covariance (ANCOVA) (13/114); logistic regression was applied for binary and ordinal outcomes in 14/114 trials; and the repeated measures analysis was used in 39/114 trials.

Conclusion: The majority of trials used PROs as primary and/or secondary outcomes. Conventional methods such as linear regression are widely used, despite the likely violation of their assumptions. In recent years, there is an increasing trend of using complex models (e.g. with mixed effects). Statistical methods developed to address these violations when analysing PROs, such as beta-binomial regression, are not routinely used in practice. Future research will focus on evaluating available statistical methods for the analysis of PROs.

For peer review only

Article Summary

Strengths and limitations of this study

- Patient-reported outcomes (PROs) are increasingly used as clinical outcomes, and the use of appropriate statistical methods will ensure reliable estimates are produced to support decision making.
- This review summarises the up-to-date statistical methods that have been used for the primary analysis of PROs in 114 randomised controlled trials (RCTs) published by the United Kingdom's National Institute for Health Research (NIHR) Health Technology Assessment (HTA) Journal between January 1997 and December 2020, and it is the largest comprehensive review of trials on the use of PROs and the corresponding statistical methods for the analysis of PROs to date.
- Most studies apply conventional statistical methods (e.g. linear regression) for the analysis of PROs despite the likely violation of the model assumptions; and even though specialist statistical methods have been developed to solve this problem, these methods are rarely used in practice.
- There was a lack of explicit reporting of PROs in some trials. Although assumptions have been made based on the context where some required information was not explicitly stated, it is possible the extracted data was inconsistent with researchers' intention.
- This study only included trials published in the NIHR HTA Journal as the information relating to PROs in other journals are not reported in as much detail and the HTA Journal intends to publish all NIHR funded studies, regardless of their findings.

For peer review only

INTRODUCTION

Patient-reported outcomes (PROs) are health or health-related outcomes of health interventions reported by patients themselves. PROs enable health researchers to measure, analyse and compare clinical outcomes from the patient perspective and provide clinical effectiveness outcomes to support decision making. The descriptive and scoring system of a PRO can transform subjective descriptions of health to numerical scores in a range of dimensions;[1] and this transformation quantifies and allows the statistical analysis of these health-related outcomes.

Randomised controlled trials (RCTs) are regarded as the gold standard for evaluating the effectiveness of interventions.[2,3] The randomisation process in a well-conducted RCT can reduce the selection bias and allocation bias, and inform the causality of the treatment on responses.[4] These traits of RCTs can simplify the analysis of PRO data.

However, it still can be complex to analyse PRO data for the following reasons. First, one PRO can generate multiple outcomes and can be reported in different forms – using different score types such as subscales or summary scores, generating quality-adjusted survivals (i.e. the integration of the quality-of-life function over the observed period of survival, such as quality-adjusted life years), producing dichotomised outcomes (e.g. proportion of responders with a PRO score above or below a particular pre-specified value or cut-off), and producing ordinal outcomes using several cut-offs. The various forms can result in multiple endpoints, and potentially increase the complexity of analysing PRO data. Second, PRO data is likely to be bounded, discrete and skewed,[5] and when analysing PRO data using a general linear model (including *t*-test, ANOVA, ANCOVA and linear regression), the Normality assumption of residuals is likely to be violated.[6] Also, the application of statistical methods might vary according to the type of data distribution and the aim of the statistical analysis, but these features of PROs data may obscure the assignment of a PRO score to a type of classical distribution, and therefore make it difficult to decide what statistical methods need to be applied for the data analysis.

An inappropriate statistical analysis of PROs can result in unreliable estimands of clinical effectiveness and accordingly fail to provide accurate and robust results for decision-making, with wider confidence intervals or larger errors. [7,8] For example, patients may fail to receive effective treatment because this treatment is falsely shown not to be clinically effective based on inaccurate estimands; vice-versa, patients may receive treatment which may potentially harm their health when unreliable evidence supports the use of this treatment. Thus, applying appropriate statistical methods for the analysis of PROs is crucial to reduce biases of estimands, to accurately evaluate clinical effectiveness, and to support the healthcare decision-making.

For peer review only

This study aims to firstly identify how frequently PROs have been used as primary or secondary clinical outcomes in reports of RCTs published in the United Kingdom's (UK) National Institute for Health Research (NIHR) Health Technology Assessment (HTA) Journal; and secondly, when the PRO was the primary outcome for trial, to summarise the statistical methods used to analyse the outcome.

METHODS

Definition of a Patient-Reported Outcome (PRO)

There are several definitions of a PRO. For the purpose of this review, a patient-reported outcome measure (PROM) was defined as a questionnaire that measures health or a health-related outcome as a result of health interventions reported by patients themselves without any interpretation by clinicians or any other proxies. A patient-reported outcome (PRO) is an umbrella term for outcomes used to measure patients' perceptions of health-related quality of life (HRQoL), broadly quality of life (QoL), health status, satisfaction with the treatment and health conditions, etc.[1,9–11]

Trial Identification

Reports of RCTs published in the UK's NIHR HTA Journal between 1 January 1997 and 31 December 2020 that defined and reported a PRO as clinical endpoints or outcomes for the trial were systematically identified and reviewed. The HTA Journal was chosen because, in comparison to major medical journals, the information related to the trial and the PROs are reported in more detail. Information related to the use of PROs included the frequency of using PROs as clinical outcomes; whether the PROs were used as primary and/or secondary outcomes; the characteristics of the PROs when they were used as primary outcomes; and when the PRO was the primary outcome, the statistical methods used for the analysis of the PRO data. The identification of HTA reports of RCTs used the same search strategy as previous work.[12] The selection of trials with PROs was conducted by one reviewer (YQ). Three reviewers (SW, RJ, and LF) conducted quality assurance checks on 30% of the included papers after the data extraction was completed, and disagreements were discussed to achieve consensus.

Inclusion and exclusion criteria

The studies included in this review satisfied the following criteria: 1) individually randomised controlled trials; 2) trials with at least one PRO as the primary outcome; and 3) trials with the statistical analysis conducted for the PRO. Studies excluded from this review included cluster RCTs as these have specific statistical issues; influenza trials as these rarely use PROs as

For peer review only

clinical outcomes; adaptive or group sequential trials as these have different statistical issues that may influence the choice of analysis; follow-on studies and pilot studies.

PROs identified in this review can be well-established measures from previous studies with feasibility, reliability and validity tested, or self-developed measures by researchers alongside trials. For studies with measures which were not clearly defined as a PRO in the trial, various methods were taken to identify whether the measure was categorised as a PRO, including retrieving the cited paper that developed the measure, identifying signal words such as 'carers' and 'physicians' for rating or assessing patients' outcomes in the measure description, and referring to other papers that developed or applied the outcome measure. According to our definition of PROs, trials that only recruited patients, or trials that recruited both patients and proxies when patients were unable to complete PROs were included; trials that only used proxies as informants to complete the PROs were excluded in order to avoid the cases where clinicians respond to health outcome measures on the patients' behalf.

Trials using the product of PROs, such as a dichotomised outcome and quality-adjusted survivals, were included. Trials that used PROs only as primary cost-effectiveness outcomes, but not as clinical primary outcomes were excluded. Even if the statistical methods were proposed, trials that did not actually conduct the statistical analysis were excluded.

Data Extraction

The following information was extracted from the included trials:

1. Characteristics of the trials with PROs as primary outcomes, including the number of participants randomised and analysed, the baseline and post-randomisation assessment, the most frequently used PROs, and special types of PROs (including patient satisfaction, preference-based and proxy-reported).
2. Statistical methods conducted for the primary analysis of the PROs, including the study population, the specific statistical methods, the adjustment for baseline score or other covariates, involvement of random effects, robust standard errors and bootstrapping techniques, repeated measures analysis, and strategies for missing data.
3. The quality of reporting PROs, including whether there is a clear definition or justification of the primary outcomes, primary endpoints, statistical methods, and covariates.

For the purpose of this review, the statistical methods were broadly categorised into two categories: 'univariable methods' that do not adjust for any other covariates except the randomised group (e.g. *t*-test, Chi-squared test, and simple linear regression) and 'multivariable methods' that have one or more explanatory variables (e.g. baseline score) in addition to the

For peer review only

randomised treatment group (e.g. multiple linear regression). The multivariable methods were further classified according to the categories of generalized linear models (GzLM), including linear regression, analysis of covariance (ANCOVA), binary logistic regression, ordinal logistic regression, and their extensions for correlated responses such as coefficients estimated by generalised estimating equations (GEEs) and mixed effect models. Repeated measures analysis for PROs with more than one post-baseline assessments were classified into four categories: response feature analysis (i.e. using summary measures, such as the area under the curve or post-randomisation mean score), generalized linear mixed models (GzLMM), GzLM with parameters estimated by GEE, and repeated measures analysis of variance (ANOVA).[5,13]

Patient and Public Involvement

Patients and the public were not involved in any way in this study.

RESULTS

In total, 1356 reports were published by the HTA Journal between 1 January 1997 and 31 December 2020, and 940 reports were excluded after screening the titles and abstracts. In the remaining 416 reports, 115 were excluded for various reasons (Figure 1). In the 303 published individually randomised controlled trials, 37.9% (114/303) of trials used PROs as primary outcomes and 83.4% (251/303) of trials used PROs as secondary outcomes. Two trials with PROs as primary outcomes were excluded as they were closed without conducting a statistical analysis of the data using the statistical methods that were proposed in the report.[14,15] It should be noted that the first RCT with a PRO as a clinical outcome was published in the HTA Journal in 1999,[16] and the earlier reports published in the HTA Journal were mainly systematic reviews.

All included trials that used PROs as primary outcomes also used PROs as secondary outcomes. The trend of using PROs as clinical outcomes in trials between 1999 to 2020 is shown in Figure 2. Except for the earlier years (1999-2003) with a small number of studies, the proportion of trials with PROs used as secondary outcomes (the red curve) is approximately two times higher than the proportion of the trials with PROs as primary outcomes (the blue curve). Generally, there is an increase in using PROs as clinical outcomes in HTA trials.

Trial characteristics

In total, 83.1% (61,715/74,298) of the participants randomised in the 114 trials were used for the primary analysis (Table 1). The characteristics of these trials are summarised in Table 2. The most common design was two arm parallel group trial. More than a half trials were in either mental health (30/114) or musculoskeletal conditions (28/114). Most trials collected baseline

For peer review only

assessments (101/114) and more than one post-baseline assessments (107/114). The maximum number of post-baseline assessment was 24 in a trial on eczema management for children.[17]

Most trials (107/114) clearly defined the primary outcomes; the sample size calculation implied the primary outcomes for the trial was a PRO in six trials that did not explicitly specify the primary outcome, and one trial defined PROs as main outcome measures but used an alternative outcome for the sample size calculation.[18] Table 3 summarises the PROs used as primary outcomes in three or more included trials. The most popular PROs were mainly generic (i.e. SF-36/SF-6D and EQ-5D) and depression-specific (i.e. BDI, HADS and PHQ). Eight trials used more than one PRO as the primary outcomes, and fourteen trials used non-PRO clinical outcomes as co-primary outcomes.

Preference-based PROs were used in six trials as primary outcomes, including five[32–36] that used the EQ-5D and one[37] that used the SF-6D. Seven trials used the quality-adjusted survivals, including three [33,34,36] that used EQ-5D and four[38–41] that used specific PROs for estimation. Patient satisfaction was used as primary outcome in two trials.[42,43] Proxies were recruited in six trials[17,44–48] that primarily aimed to collect patient-reported outcomes, and only recruited proxies for patients who were unable to complete the PROs. Health outcomes assessed by investigators were not included as primary outcomes, e.g. four trials using Quality of Life Scale or Rankin Scale that were completely assessed by investigators were excluded.[49–51]

Different score types were used to summarise the PRO primary outcomes. Most trials (98/114) used one score type to report the primary outcome, and the rest trials used two types of scores. Summary scores were the most popular way to measure PROs (83/114), followed by subscales (30/114) and single items (16/114), including Likert scale (11/114) and visual analogue scale (VAS) (5/114).

Table 1 Recruitment and retention of trial participants included in the review

Items	Mean	Median	SD	Min	Max	Total
Number of participants randomised	652	480	928	85	8,003	74,298
Number of participants analysed*	541	388	847	65	7,677	61,715

*number of participants analysed in the primary analysis of PROs; if multiple post-baseline assessments were used for primary outcome, the number of participants analysed at the longest primary endpoint was taken. SD, standard deviation.

For peer review only

Table 2 Trial design and assessments characteristics of the trials included in the review

Items		No	%	Total
Trial design				114
	Parallel group	102	87.2	
	Factorial	3	2.6	
	Crossover	0	0.0	
	Other*	9	7.7	
Number of arms				114
	2	83	70.9	
	3	21	17.9	
	4	5	4.3	
	>4	5	4.3	
Clinical area				114
	Mental Health	30	25.6	
	Musculoskeletal	28	23.9	
	Obstetrics and gynaecology	9	7.7	
	Gastrointestinal	7	6.0	
	Respiratory	5	4.3	
	Stroke	5	4.3	
	Primary Care	4	3.4	
	Cardiovascular	4	3.4	
	Cancer/Oncology	3	2.6	
	Dermatology	4	3.4	
	Other^	15	12.8	
Number of trials with a baseline assessment of the PRO				114
		101	86.3	
Timing of primary outcome post-baseline assessments				114
	<1 month	7	6.0	
	1-6 months	28	23.9	
	6-18 months	50	42.7	
	>18 months	27	23.1	
	Missing$	2	1.7	
Number of post-baseline assessments				114
	1	5	4.3	
	2	41	35.0	
	3	29	24.8	
	4	19	16.2	
	>4	18	15.4	
	Missing$	2	1.7	

*patient preference/Zelen's.

^chronic fatigue, minor surgery, multiple sclerosis, neurosurgery, paediatric, sleep disorders, urology, vascular.

$Two trials did not specify the timing and number of post-baseline assessments.

Table 3 The most frequently used PROs as primary outcomes in the included trials

Patient-Reported Outcomes	Abbr.	No	%	Ref
Short Form-36<br>Short Form 6-Dimension	SF-36<br>SF-6D	8	7.0	[21–24]
Beck Depression Inventory	BDI	7	6.1	[25]
Hospital Anxiety and Depression Scale	HADS	5	4.4	[26]
EuroQol-5 Dimension	EQ-5D	5	4.4	[27–29]
Patient Health Questionnaire	PHQ	5	4.4	[30]
Oxford Shoulder Score	OSS	4	3.5	[31]
Other		90	78.9	
Total*		124	108.8	

* The total number of included trials is 114. Eight trials used more than one PRO as primary outcomes, including two trials that used three PROs, and six trials that used two PROs as primary outcomes.

Statistical methods for the primary analysis of PROs

Intention-to-treat (ITT) analysis (i.e. analysis based on the treatment assignment of all participants but not the actual treatment received)[52], including ITT with and without missing data imputation, was used in 111/114 trials, and 46 trials used other study populations such as per protocol analysis (i.e. analysis based on the patients who completed the originally treatment assigned), as treated, or complier average causal effect analysis (i.e. analysis of the treatment effect based on the subgroup that completed the originally treatment assigned) for the secondary or sensitivity analysis.

The statistical methods applied for primary analyses were clearly defined in 79/114 trials, and the use of univariable or multivariable methods for primary analyses were justified in 92/114 trials. Except for two trials that did not specify the timing and number post-randomisation assessments,[19,20] 72/114 clearly defined the single timepoint used for the primary analysis (e.g. x-months post-baseline), and 40/114 used the repeated post-baseline outcomes for the primary analysis.

The statistical methods used for the primary analysis of PROs are shown in Table 4. The linear mixed model (45/114) and linear regression (29/114) and ANCOVA (13/114) were the most popular methods. Among the 45 trials using linear mixed models for the primary analysis, 23 trials conducted a repeated measures analysis, and the rest did not consider the repeated post-baseline outcomes in the primary analysis. Three trials used GzLM with coefficients estimated by GEE for the longitudinal primary analysis, including one as an extension to ordinal logistic regression[53] and two as an extension to linear regression.[49,54] Only one trial with quality-adjusted survivals used Cox regression for the primary analysis.[36]

Bootstrapped confidence intervals were calculated after using a general linear model (including *t*-test, ANCOVA, linear regression) or linear mixed model in six trials[16,18,33,48,55,56] due

For peer review only

to the skewness of PRO scores, and one additional trial did not conduct any statistical analysis except calculating the bootstrapped confidence interval[57]. Robust standard errors can be used to estimate confidence intervals and the calculation of test statistics (and associated P-values). Six trials[48,53,58–60] reported using robust standard errors based on regression methods for the primary analysis, and three trials[54,61,62] reported using robust standard errors for the longitudinal analysis in the non-primary analysis.

There were 106 trials that used multivariable methods, of which (98/106) clearly reported the covariates adjusted in the primary analysis. Baseline values of the PRO were used to adjust analyses in 85 trials, and three trials[63–65] modelled the change of PRO from baseline in the primary analysis. The use of random effects for the primary analysis of PROs were clearly specified in 47 trials: 44 used linear mixed models, and the rest three used repeated measures ANOVA [66], binary logistic mixed model[38], and ordinal logistic mixed model[43] respectively. The most common random factors applied in the multivariable methods were therapists, centres (i.e. hospital sites), and individual patients.

The majority of trials stated the proportion of missing PRO data (109/114), developed strategies to deal with missing data (99/114), imputed missing data using various methods such as mean imputation and last observation carried forward (89/114). In 40/114 studies, missing data were imputed as part of a sensitivity analysis to check the robustness of the primary analysis strategy which did not consider missing data.

Trend of using statistical methods for the primary analysis of PROs

Figure 3 shows the trend of using different statistical methods over the observed period. There is an increasing trend for using more complex models (mixed models and GLzM with GEE) in most recent years for the analysis of PROs. The linear regression, ANCOVA and repeated measures ANOVA were the most popular regression methods used from 1999 to 2010, used in around 63% of trials averagely. But this popularity dropped to 28.6% during 2011-2015 and 30.0% during 2016-2020. In contrast, the proportion of trials using the linear mixed model and linear regression with GEE for correlated outcomes increased across the observation period, from 11.1% to 54.0%. While the use of binary logistic regression slightly decreased from 11.1% to 4.0% over time, the proportion of trials using ordinal logistic regression for the primary analysis remained small.

For peer review only

Table 4 Statistical methods for the main or primary analysis of PROs that are the primary outcomes of the trials

Statistical methods	N	%	Total
Univariable methods			27
t-test	11	40.7	
unadjusted regression methods*	7	25.9	
Chi-squared test	3	11.1	
Wilcoxon rank-sum test (Mann–Whitney U test)	4	14.8	
Kruskal-Wallis test	1	3.7	
log-rank test	1	3.7	
Multivariable methods^			106
linear mixed model	45	42.5	
linear regression	28	27.4	
ANCOVA	13	12.3	
linear regression with GEE	2	1.9	
binary logistic regression	8	7.5	
binary logistic mixed model	1	0.9	
ordinal logistic regression	4	3.8	
ordinal logistic mixed model	1	0.9	
repeated measures ANOVA	6	5.7	
survival analysis	1	0.9	
Repeated measures analysis			39
linear mixed model	23	59.0	
response feature analysis$	7	17.9	
repeated measures ANOVA	6	15.4	
GzLM with GEE	3	7.7	

*In seven trials using unadjusted regression methods, three trials used linear regression, one used linear mixed model, two used ordinal logistic regression and one used binary logistic regression.

^106 trials used multivariable methods for the analysis of PROs, including four trials that used two different methods for the primary analysis of PROs.

$In seven trials using response feature analysis (with quality-adjusted survivals) in primary analysis, four used linear regression or ANCOVA, two used linear mixed models, and one used survival analysis.

ANCOVA, analysis of covariance; ANOVA, analysis of variance; GzLM, generalised linear model; MLE, maximum likelihood estimation; GEE, generalised estimating equation.

DISCUSSION

This study has systematically conducted a review which summarised how frequently PROs have been used and what statistical methods have been applied for the primary analysis of PROs in RCTs published by the UK's NIHR HTA Journal between 1997 and 2020. This review found that 83.4% (251/303) used PROs as primary or secondary outcome, and 37.9% (114/303) used PROs as primary outcomes. Though conventional methods (such as the *t*-test and multiple linear regression & ANCOVA) have been widely used, there is a growing trend of using more complex methods such as linear mixed models (with both fixed and random effects) for the analysis of PROs over the observed period.

The general linear model (including *t*-test, ANOVA, ANCOVA and linear regression) and linear mixed model were widely used over the past two decades, which could possibly result from the frequent use of continuous data type of response variables. The trend of using complex models could result from the increasingly complexity of trial designs and from recommendations on using linear mixed models over repeated measures ANOVA for the longitudinal analysis of PROs.[5,67] There is a decrease in the use of binary logistic regression as the dichotomised outcome gathers less information from the PROs compared to other score types.[68]

There is a gap between statistical methods developed by methodologists and statistical methods used for the analysis of PROs in trials. For example, Arostegui, Núñez-Antón and Quintana recommend the use of ordinal logistic regression with random effects model, beta-binomial regression or beta-logit-normal regression for continuous or ordinal PRO data after testing distributional assumptions.[67,69] However, only one of the five included trials that used ordinal logistic regression for the primary analysis considered random effects.[43] Nither beta-binomial regression nor beta-logit-normal regression were used by the 114 trials. Interestingly, the international standards for the analysis of PROs in cancer trials by the SISAQOL Consortium neither recommended the use of ordinal regression nor compared ordinal regression with other statistical methods.[70] The conventional methods are popular since the estimands produced by simple models can remain robust regardless of the violation of model assumptions.[6] In addition, the difficulty in applying newly developed models in statistical software and the complexity to explain estimands of these models may make researchers more reluctant to use them.

To the best of our knowledge this study is by far the largest review of trials (with 114 studies) published by the HTA journal which analysed the frequency of using PROs and the statistical methods for the analysis of PROs. The reviews by Pe et al (2018) of trials in breast cancer; Hamel et al (2017) (lung cancer), and Fiteni et al 2016 (lung cancer) had sample sizes of 66,

For peer review only

33, 27 articles respectively.[7,8,71] Compared to other reviews that concentrate on only one disease area (i.e. oncology),[7,8,71–74] this review summarised the details in the frequency of using PROs and applying statistical methods in RCTs with a range of disease areas.

This study has the following limitations. First, this review only looked at publicly funded trials in the UK. It is possible that statistical methods are used differently in industry funded trials or in trials in other countries. However, as the NIHR HTA Journal intends to publish all NIHR funded projects, it has less publication bias compared to journals that only publish positive outcomes, and the information related to PROs in other journals are not reported in as much detail. The extracted statistical methods for the analysis of PROs from this review are consistent with those included from other similar reviews.[7,8,71–74] Second, there might be other appropriate methods for the analysis of PROs that were not included in this review. This review mainly analysed RCTs with PROs as primary outcomes because primary outcomes and the corresponding statistical methods were more explicitly reported.

Third, trials with PROs only used as cost-effectiveness outcomes were excluded. This is because the statistical strategies for clinical effectiveness and cost-effectiveness outcomes may vary, and cost-effectiveness analysis (CEA) produces both cost and clinical effectiveness outcomes. If PROs for CEA were included in this review, the proportion of included trials would increase, as there were some studies using EQ-5D for the primary CEA. It could be argued that the analysis of effectiveness estimated by PROs in CEA also requires appropriate statistical methods, but estimands calculated for a health economic analysis could be different from those for a clinical analysis as they hold different purposes to conduct these analyses. Therefore, we believe it is justified to make this exclusion.

Last, the information related to PROs and statistical methods were not clearly reported in some trials. Although assumptions have been made based on the context where some required information was not explicitly stated, it is possible that the data extracted was inconsistent with researchers' intention. However, as the data have been extracted for all reports by one reviewer there is consistency in the interpretation and assumptions made.

To produce explicit reports, it is recommended that researchers follow specific guidelines that can instruct the reporting of using PROs in RCT papers and protocols such as the CONSORT PRO Extension,[11] SPIRIT PRO Extension,[75] and the standards for the analysis of PROs in cancer RCTs.[70] In addition, a clear classification of the terminology of the statistical methods is desired. It is a historical problem that the names of statistical methods are confusing (e.g. general linear model vs. generalized linear model), and multiple terms can be used to describe the same method, for example, the proportional odds model, ordered logit model and ordinal

For peer review only

logistic regression refer to the same regression technique. Thus, researchers should be clear and cautious when describing the exact statistical method for the analysis.

In conclusion, the majority of trials funded by the NIHR HTA Programme used PROs as primary and/or secondary outcomes. Although there is an increasing trend of using complex models (e.g. mixed effects), conventional methods such as linear regression remain widely used for the analysis of PROs, despite the likely violation of their assumptions. Statistical methods developed to address these violations when analysing PROs, such as beta-binomial regression, are not routinely used in practice. Various methods for the analysis of PROs have been identified from this review, but it is still unknown which methods are the most appropriate for the analysis of PRO data. Future research will focus on evaluating available statistical methods and make recommendations on using different methods for the analysis of PRO data.

For peer review only

Figure Legends

Figure 1 Flow diagram for the inclusion and exclusion of trials published in the Health Technology Assessment Journal from 1997 to 2020

Figure 2 Number and proportion of trials using PROs as primary and/or secondary outcomes from 1999 to 2020

Figure 3 The percentage of trials using multivariable methods for the primary analysis of PROs from 1999 to 2020

(N = ?) denotes the number of trials published in the specified period. As the survival analysis was only used in one trial, it is not shown in this graph. As this graph only summarised multivariable methods and one trial could use two or more multivariable methods for the primary analysis, the number of trials summarised in this graph may not equal the total number of included trials.

For peer review only

Contributors All authors contributed to the study concept and study design. YQ and RJ contributed to the selection of data. YQ conducted the data extraction and data analysis. SW, RJ, and LF contributed to the quality assurance check of the data. YQ drafted the manuscript. All authors critically revised the manuscript and approved the final manuscript.

Competing interests The PhD study of Qian is financially sponsored by the University of Sheffield and China Scholarship Council (Grant No. 201908890049). Prof. Walters, Dr. Jacques, and Dr. Flight received funding across various projects by NIHR. Prof. Walters is a National Institute for Health Research (NIHR) Senior Investigator (NF-SI-0617-10012) supported by the NIHR for this research project. The views expressed in this publication are those of the author(s) and not necessarily those of the NIHR, NHS or the UK Department of Health and Social Care. These organisations had no role in the study design; in the collection, analysis, and interpretation of the data; in the writing of the report; or in the decision to submit the paper for publication.

Funding Qian is sponsored jointly by the University of Sheffield and China Scholarship Council (Grant No. 201908890049). Prof. Walters, Dr. Jacques, and Dr. Flight received funding across various projects by NIHR. Prof. Walters is a National Institute for Health Research (NIHR) Senior Investigator (NF-SI-0617-10012) supported by the NIHR for this research project. The views expressed in this publication are those of the author(s) and not necessarily those of the NIHR, NHS or the UK Department of Health and Social Care. These organisations had no role in the study design; in the collection, analysis, and interpretation of the data; in the writing of the report; or in the decision to submit the paper for publication.

Patient consent for publication This review does not involve recruiting new participants or analysing individual participants data. Individual informed consent was obtained to take part in the original trials by primary investigators.

Data availability statement The information extracted in this review is based on published trials in the Health Technology Assessment Journal. The data extracted from the HTA Journal supporting the finding of this study is available upon reasonable request from Yirui Qian at yqian21@sheffield.ac.uk.

Ethical statement: The information extracted in this review is based on published HTA trials where ethics approvals were obtained by the original trial teams. This review does not involve recruiting new participants or analysing individual participants, and the original participants cannot be identified from this review.

For peer review only

REFERENCES

1 Brazier J, Ratcliffe J, Salomon JA, *et al.* Introduction to the measurement and valuation of health. In: *Measuring and Valuing Health Benefits for Economic Evaluation (2 ed.)*. 2016. 7–30. doi:10.1093/med/9780198725923.001.0001

2 Akobeng AK. Understanding randomised controlled trials. *Arch. Dis. Child.* 2005;**90**:840–4. doi:10.1136/adc.2004.058222

3 Altman DG. Better reporting of randomised controlled trials: The CONSORT statement. *BMJ*. 1996;**313**:570–1. doi:10.1136/bmj.313.7057.570

4 Moher D, Hopewell S, Schulz KF, *et al.* CONSORT 2010 explanation and elaboration: updated guidelines for reporting parallel group randomised trials. *BMJ* 2010;**340**. doi:10.1136/bmj.c869

5 Walters SJ. *Quality of Life Outcomes in Clinical Trials and Health-Care Evaluation*. Chichester, UK: : John Wiley & Sons, Ltd 2009. doi:10.1002/9780470840481

6 Walters SJ, Campbell MJ. The use of bootstrap methods for analysing health-related quality of life outcomes (particularly the SF-36). *Health Qual Life Outcomes* 2004;**2**. doi:10.1186/1477-7525-2-70

7 Hamel JF, Saulnier P, Pe M, *et al.* A systematic review of the quality of statistical methods employed for analysing quality of life data in cancer randomised controlled trials. *Eur. J. Cancer*. 2017;**83**:166–76. doi:10.1016/j.ejca.2017.06.025

8 Pe M, Dorme L, Coens C, *et al.* Statistical analysis of patient-reported outcome data in randomised controlled trials of locally advanced and metastatic breast cancer: a systematic review. *Lancet Oncol* 2018;**19**:e459–69. doi:10.1016/S1470-2045(18)30418-2

9 Fitzpatrick R, Davey C, Buxton MJ, *et al.* Evaluating patient-based outcome measures for use in clinical trials. *Health Technol Assess (Rockv)* 1998;**2**. doi:10.3310/hta2140

10 Mokkink LB, Terwee CB, Patrick DL, *et al.* The COSMIN checklist for assessing the methodological quality of studies on measurement properties of health status measurement instruments: An international Delphi study. *Qual Life Res* 2010;**19**:539–49. doi:10.1007/s11136-010-9606-8

11 Calvert M, Blazeby J, Altman DG, *et al.* Reporting of patient-reported outcomes in randomized trials: The CONSORT PRO extension. *J. Am. Med. Assoc.* 2013;**309**:814–22. doi:10.1001/jama.2013.879

For peer review only

12 Walters SJ, Dos Anjos Henriques-Cadby IB, Bortolami O, *et al.* Recruitment and retention of participants in randomised controlled trials: A review of trials funded and published by the United Kingdom Health Technology Assessment Programme. *BMJ Open* 2017;**7**:15276. doi:10.1136/bmjopen-2016-015276

13 Schober P, Vetter TR. Repeated measures designs and analysis of longitudinal data: If at first you do not succeed-try, try again. *Anesth Analg* 2018;**127**:569–75. doi:10.1213/ANE.0000000000003511

14 Williams NH, Jenkins A, Goulden N, *et al.* Subcutaneous injection of Adalimumab Trial compared with Control (SCIATiC): A randomised controlled trial of adalimumab injection compared with placebo for patients receiving physiotherapy treatment for sciatica. *Health Technol Assess (Rockv)* 2017;**21**:1–179. doi:10.3310/hta21600

15 Mihaylov S, Stark C, McColl E, *et al.* Stepped treatment of older adults on laxatives. The STOOL trial. *Health Technol Assess (Rockv)* 2008;**12**. doi:10.3310/hta12130

16 Morrell CJ, Spiby H, Stewart P, *et al.* Costs and benefits of community postnatal support workers: A randomised controlled trial. *Health Technol Assess (Rockv)* 2000;**4**. doi:10.3310/hta4060

17 Santer M, Rumsby K, Ridd MJ, *et al.* Adding emollient bath additives to standard eczema management for children with eczema: The BATHE RCT. *Health Technol Assess (Rockv)* 2018;**22**:1–116. doi:10.3310/hta22570

18 Kerry S, Hilton S, Patel S, *et al.* Routine referral for radiography of patients presenting with low back pain: Is patients' outcome influenced by GPs' referral for plain radiography? *Health Technol Assess (Rockv)* 2000;**4**. doi:10.3310/hta4200

19 Little P, Hobbs FDR, Moore M, *et al.* PRImary care Streptococcal Management (PRISM) study: In vitro study, diagnostic cohorts and a pragmatic adaptive randomised controlled trial with nested qualitative study and cost-effectiveness study. *Health Technol Assess (Rockv)* 2014;**18**:1–101. doi:10.3310/hta18060

20 Little P, Turner S, Rumsby K, *et al.* Dipsticks and diagnostic algorithms in urinary tract infection: Development and validation, randomised trial, economic analysis, observational cohort and qualitative study. *Health Technol Assess (Rockv)* 2009;**13**. doi:10.3310/hta13190

21 J E Ware Jr, C D Sherbourne. The MOS 36-item short-form health survey (SF-36). I. Conceptual framework and item selection - PubMed. *Med Care* 1992;**30**:473–83.https://pubmed.ncbi.nlm.nih.gov/1593914/ (accessed 8 Mar 2021).

For peer review only

22 McHorney CA, Ware JE, Raczek AE. The MOS 36-item short-form health survey (Sf-36): II. Psychometric and clinical tests of validity in measuring physical and mental health constructs. *Med Care* 1993;**31**:247–63. doi:10.1097/00005650-199303000-00006

23 McHorney CA, Ware JE, Rachel Lu JF, *et al.* The MOS 36-item short-form health survey (SF-36): III. Tests of data quality, scaling assumptions, and reliability across diverse patient groups. *Med Care* 1994;**32**:40–66. doi:10.1097/00005650-199401000-00004

24 Brazier J, Roberts J, Deverill M. The estimation of a preference-based measure of health from the SF-36. *J Health Econ* 2002;**21**:271–92. doi:10.1016/S0167-6296(01)00130-8

25 Beck AT, Steer RA, Carbin MG. Psychometric properties of the Beck Depression Inventory: Twenty-five years of evaluation. *Clin Psychol Rev* 1988;**8**:77–100. doi:10.1016/0272-7358(88)90050-5

26 Zigmond AS, Snaith RP. The Hospital Anxiety and Depression Scale. *Acta Psychiatr Scand* 1983;**67**:361–70. doi:10.1111/j.1600-0447.1983.tb09716.x

27 The EuroQol Group. EuroQol - a new facility for the measurement of health-related quality of life. *Health Policy (New York)* 1990;**16**:199–208. doi:10.1016/0168-8510(90)90421-9

28 Dolan P. Modeling Valuations for EuroQol Health States. *Med Care* 1997;**35**:1095–108. doi:10.1097/00005650-199711000-00002

29 Herdman M, Gudex C, Lloyd A, *et al.* Development and preliminary testing of the new five-level version of EQ-5D (EQ-5D-5L). *Qual Life Res* 2011;**20**:1727–36. doi:10.1007/s11136-011-9903-x

30 Spitzer RL, Kroenke K, Williams JBW. Validation and utility of a self-report version of PRIME-MD: The PHQ Primary Care Study. *J Am Med Assoc* 1999;**282**:1737–44. doi:10.1001/jama.282.18.1737

31 Dawson J, Fitzpatrick R, Carr A. Questionnaire on the perceptions of patients about shoulder surgery. *J Bone Jt Surg - Ser B* 1996;**78**:593–600. doi:10.1302/0301-620x.78b4.0780593

32 Gazzard G, Konstantakopoulou E, Garway-Heath D, *et al.* Selective laser trabeculoplasty versus drops for newly diagnosed ocular hypertension and glaucoma: The LiGHT RCT. *Health Technol Assess (Rockv)* 2019;**23**:1–101.

For peer review only

doi:10.3310/hta23310

33 Sharples L, Everett C, Singh J, *et al.* Amaze: A double-blind, multicentre randomised controlled trial to investigate the clinical effectiveness and cost-effectiveness of adding an ablation device-based maze procedure as an adjunct to routine cardiac surgery for patients with pre-existing atrial fibrillation. *Health Technol Assess (Rockv)* 2018;**22**. doi:10.3310/hta22190

34 Watson AJM, Cook J, Hudson J, *et al.* A pragmatic multicentre randomised controlled trial comparing stapled haemorrhoidopexy with traditional excisional surgery for haemorrhoidal disease: The eTHoS study. *Health Technol Assess (Rockv)* 2017;**21**:1–223. doi:10.3310/hta21700

35 Brittenden J, Cotton SC, Elders A, *et al.* Clinical effectiveness and cost-effectiveness of foam sclerotherapy, endovenous laser ablation and surgery for varicose veins: Results from the comparison of LAser, Surgery and foam Sclerotherapy (CLASS) randomised controlled trial. *Health Technol Assess (Rockv)* 2015;**19**:1–341. doi:10.3310/hta19270

36 Russell I, Edwards R, Gliddon A, *et al.* Cancer of oesophagus or gastricus: New assessment of technology of endosonography (COGNATE): Report of pragmatic randomised trial. *Health Technol Assess (Rockv)* 2013;**17**:1–13. doi:10.3310/hta17390

37 Michaels JA, Campbell WB, Brazier JE, *et al.* Randomised clinical trial, observational study and assessment of cost-effectiveness of the treatment of varicose veins (REACTIV trial). *Health Technol Assess (Rockv)* 2006;**10**:1–114. doi:10.3310/hta10130

38 Pickard R, Goulao B, Carnell S, *et al.* Open urethroplasty versus endoscopic urethrotomy for recurrent urethral stricture in men: The open rct. *Health Technol Assess (Rockv)* 2020;**24**:1–110. doi:10.3310/hta24610

39 Hewison J, Nixon J, Fountain J, *et al.* Amniocentesis results: Investigation of anxiety. The ARIA trial. *Health Technol Assess (Rockv)* 2006;**10**. doi:10.3310/hta10500

40 Bedson E, Bell D, Carr D, *et al.* Folate augmentation of treatment - Evaluation for depression (folated): Randomised trial and economic evaluation. *Health Technol Assess (Rockv)* 2014;**18**:1–159. doi:10.3310/hta18480

41 Williams JG, Alam MF, Alrubaiy L, *et al.* Comparison Of iNfliximab and ciclosporin in STeroid Resistant Ulcerative Colitis: Pragmatic randomised trial and economic evaluation (CONSTRUCT). *Health Technol Assess (Rockv)* 2016;**20**:1–322. doi:10.3310/hta20440

For peer review only

42 Townsend J, Wolke D, Hayes J, *et al.* Routine examination of the newborn: The EMREN study. Evaluation of an extension of the midwife role including a randomised controlled trial of appropriately trained midwives and paediatric senior house officers. *Health Technol Assess (Rockv)* 2004;**8**. doi:10.3310/hta8140

43 Cooper K, Breeman S, Scott NW, *et al.* Laparoscopic supracervical hysterectomy compared with second-generation endometrial ablation for heavy menstrual bleeding: The HEALTH RCT. *Health Technol Assess (Rockv)* 2019;**23**. doi:10.3310/hta23530

44 Dennis M, Forbes J, Graham C, *et al.* Fluoxetine to improve functional outcomes in patients after acute stroke: The focus rct. *Health Technol Assess (Rockv)* 2020;**24**:1–94. doi:10.3310/hta24220

45 Dennis M, Lewis S, Cranswick G, *et al.* FOOD: A multicentre randomized trial evaluating feeding policies in patients admitted to hospital with a recent stroke. *Health Technol Assess (Rockv)* 2006;**10**:1–91. doi:10.3310/hta10020

46 Banerjee S, Hellier J, Romeo R, *et al.* Study of the use of antidepressants for depression in dementia: The HTA-SADD trial- A multicentre, randomised, double-blind, placebo-controlled trial of the clinical effectiveness and cost-effectiveness of sertraline and mirtazapine. *Health Technol Assess (Rockv)* 2013;**17**:1–43. doi:10.3310/hta17070

47 Francis NA, Ridd MJ, Thomas-Jones E, *et al.* A randomised placebo-controlled trial of oral and topical antibiotics for children with clinically infected eczema in the community: The ChildRen with eczema, antibiotic management (CREAM) study. *Health Technol Assess (Rockv)* 2016;**20**. doi:10.3310/hta20190

48 Weindling AM, Cunningham CC, Glenn SM, *et al.* Additional therapy for young children with spastic cerebral palsy: A randomised controlled trial. *Health Technol Assess (Rockv)* 2007;**11**. doi:10.3310/hta11160

49 Lewis SW, Davies L, Jones PB, *et al.* Randomised controlled trials of conventional antipsychotic versus new atypical drugs, and new atypical drugs versus clozapine, in people with schizophrenia responding poorly to, or intolerant of, current drug treatment. *Health Technol Assess (Rockv)* 2006;**10**:1–94. doi:10.3310/hta10170

50 Langhorne P, Wu O, Rodgers H, *et al.* A very early rehabilitation trial after stroke (AVERT): a Phase III, multicentre, randomised controlled trial. *Health Technol Assess (Rockv)* 2017;**21**:1–119. doi:10.3310/hta21540

51 Bath PM, Woodhouse LJ, Appleton JP, *et al.* Triple versus guideline antiplatelet therapy to prevent recurrence after acute ischaemic stroke or transient ischaemic attack: The

For peer review only

TARDIS RCT. *Health Technol Assess (Rockv)* 2018;**22**:1–75. doi:10.3310/hta22480

52 Montedori A, Bonacini MI, Casazza G, *et al.* Modified versus standard intention-to-treat reporting: Are there differences in methodological quality, sponsorship, and findings in randomized trials? A cross-sectional study. *Trials* 2011;**12**:58. doi:10.1186/1745-6215-12-58

53 Kennedy TM, Chalder T, McCrone P, *et al.* Cognitive behavioural therapy in addition to antispasmodic therapy for irritable bowel syndrome in primary care: Randomised controlled trial. *Health Technol. Assess. (Rockv)*. 2006;**10**. doi:10.3310/hta10190

54 Molassiotis A, Russell W, Hughes J, *et al.* The effectiveness and cost-effectiveness of acupressure for the control and management of chemotherapy-related acute and delayed nausea: Assessment of Nausea in Chemotherapy Research (ANCHoR), a randomised controlled trial. *Health Technol Assess (Rockv)* 2013;**17**:1–114. doi:10.3310/hta17260

55 Shawo L, Bhattaraio N, Cant R, *et al.* An extended stroke rehabilitation service for people who have had a stroke: The extras rct. *Health Technol Assess (Rockv)* 2020;**24**:1–202. doi:10.3310/hta24240

56 Symmons D, Tricker K, Roberts C, *et al.* Aggressive versus symptomatic therapy in established rheumatoid arthritis. *Health Technol Assess (Rockv)* 2005;**9**.

57 Wiggins M, Oakley A, Roberts I, *et al.* The social support and family health study: A randomised controlled trial and economic evaluation of two alternative forms of postnatal support for mothers living in disadvantaged inner-city areas. *Health Technol Assess (Rockv)* 2004;**8**. doi:10.3310/hta8320

58 Kennedy ADM, Sculpher MJ, Coulter A, *et al.* A multicentre randomised controlled trial assessing the costs and benefits of using structured information and analysis of women's preferences in the management of menorrhagia. *Health Technol Assess (Rockv)* 2003;**7**. doi:10.3310/hta7080

59 Francis NA, Gillespie D, White P, *et al.* C-reactive protein point-of-care testing for safely reducing antibiotics for acute exacerbations of chronic obstructive pulmonary disease: The PACE RCT. *Health Technol Assess (Rockv)* 2020;**24**. doi:10.3310/hta24150

60 Beard DJ, Davies LJ, Cook JA, *et al.* Total versus partial knee replacement in patients with medial compartment knee osteoarthritis: The topkat rct. *Health Technol Assess (Rockv)* 2020;**24**. doi:10.3310/hta24200

For peer review only

61 Chalder M, Wiles NJ, Campbell J, *et al.* A pragmatic randomised controlled trial to evaluate the cost-effectiveness of a physical activity intervention as a treatment for depression: The treating depression with physical activity (TREAD) trial. *Health Technol Assess (Rockv)* 2012;**16**. doi:10.3310/hta16100

62 Goodyer IM, Reynolds S, Barrett B, *et al.* Cognitive-behavioural therapy and short-term psychoanalytic psychotherapy versus brief psychosocial intervention in adolescents with unipolar major depression (IMPACT): A multicentre, pragmatic, observer-blind, randomised controlled trial. *Health Technol Assess (Rockv)* 2017;**21**:1–93. doi:10.3310/hta21120

63 Goodacre S, Cohen J, Bradburn M, *et al.* The 3Mg trial: a randomised controlled trial of intravenous or nebulised magnesium sulphate versus placebo in adults with acute severe asthma. *Health Technol Assess (Rockv)* 2014;**18**. doi:10.3310/hta18220

64 Williams MA, Williamson EM, Heine PJ, *et al.* Strengthening and stretching for rheumatoid arthritis of the hand (SARAH). A randomised controlled trial and economic evaluation. *Health Technol Assess (Rockv)* 2015;**19**:221. doi:10.3310/hta19190

65 Clarke CE, Patel S, Ives N, *et al.* Clinical effectiveness and cost-effectiveness of physiotherapy and occupational therapy versus no therapy in mild to moderate Parkinson's disease: a large pragmatic randomised controlled trial (PD REHAB). *Health Technol Assess* 2016;**20**:1–96. doi:10.3310/hta20630

66 Peveler R, Kendrick T, Buxton M, *et al.* A randomised controlled trial to compare the cost-effectiveness of tricyclic antidepressants, selective serotonin reuptake inhibitors and lofepramine. *Health Technol Assess* 2005;**9**. doi:10.3310/hta9160

67 Arostegui I, Núñez-Antón V, Quintana JM. Statistical approaches to analyse patient-reported outcomes as response variables: An application to health-related quality of life. *Stat Methods Med Res* 2012;**21**:189–214. doi:10.1177/0962280210379079

68 Shields A, Coon C, Hao Y, *et al.* Patient-reported outcomes for US oncology labeling: Review and discussion of score interpretation and analysis methods. *Expert Rev Pharmacoeconomics Outcomes Res* 2015;**15**:951–9. doi:10.1586/14737167.2015.1115348

69 Arostegui I, Núñez-Antón V, Quintana JM. Analysis of the short form-36 (SF-36): The beta-binomial distribution approach. *Stat Med* 2007;**26**:1318–42. doi:10.1002/sim.2612

70 Coens C, Pe M, Dueck AC, *et al.* International standards for the analysis of quality-of-life and patient-reported outcome endpoints in cancer randomised controlled trials:

For peer review only

recommendations of the SISAQOL Consortium. *Lancet Oncol.* 2020;**21**:e83–96. doi:10.1016/S1470-2045(19)30790-9

71 Fiteni F, Anota A, Westeel V, *et al.* Methodology of health-related quality of life analysis in phase III advanced non-small-cell lung cancer clinical trials: A critical review. *BMC Cancer* 2016;**16**. doi:10.1186/s12885-016-2152-1

72 Fiteni F, Ray I Le, Ousmen A, *et al.* Health-related quality of life as an endpoint in oncology phase i trials: A systematic review. *BMC Cancer* 2019;**19**. doi:10.1186/s12885-019-5579-3

73 Nielsen LK, Abildgaard N, Jarden M, *et al.* Methodological aspects of health-related quality of life measurement and analysis in patients with multiple myeloma. *Br. J. Haematol*. 2019;**185**:11–24. doi:10.1111/bjh.15759

74 Turner-Bowker DM, Hao Y, Foley C, *et al.* The use of patient-reported outcomes in advanced breast cancer clinical trials: a review of the published literature. *Curr. Med. Res. Opin*. 2016;**32**:1709–17. doi:10.1080/03007995.2016.1205005

75 Calvert M, Kyte D, Mercieca-Bebber R, *et al.* Guidelines for inclusion of patient-reported outcomes in clinical trial protocols the spirit-pro extension. *J Am Med Assoc* 2018;**319**:483–94. doi:10.1001/jama.2017.21903

For peer review only

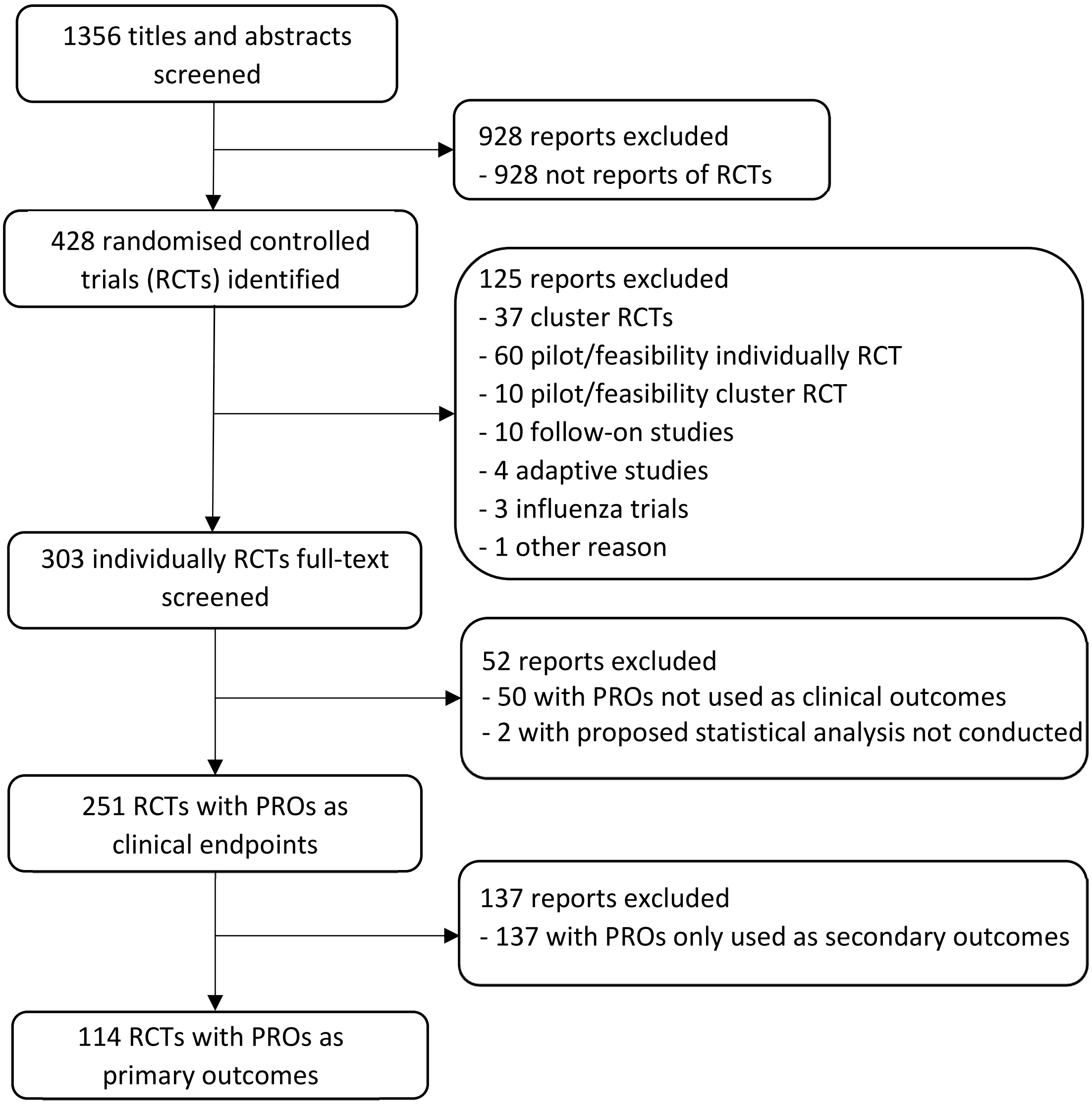
1356 titles and abstracts screened
928 reports excluded
- 928 not reports of RCTs
428 randomised controlled trials (RCTs) identified
125 reports excluded
- 37 cluster RCTs
- 60 pilot/feasibility individually RCT
- 10 pilot/feasibility cluster RCT
- 10 follow-on studies
- 4 adaptive studies
- 3 influenza trials
- 1 other reason
303 individually RCTs full-text screened
52 reports excluded
- 50 with PROs not used as clinical outcomes
- 2 with proposed statistical analysis not conducted
251 RCTs with PROs as clinical endpoints
137 reports excluded
- 137 with PROs only used as secondary outcomes
114 RCTs with PROs as primary outcomes

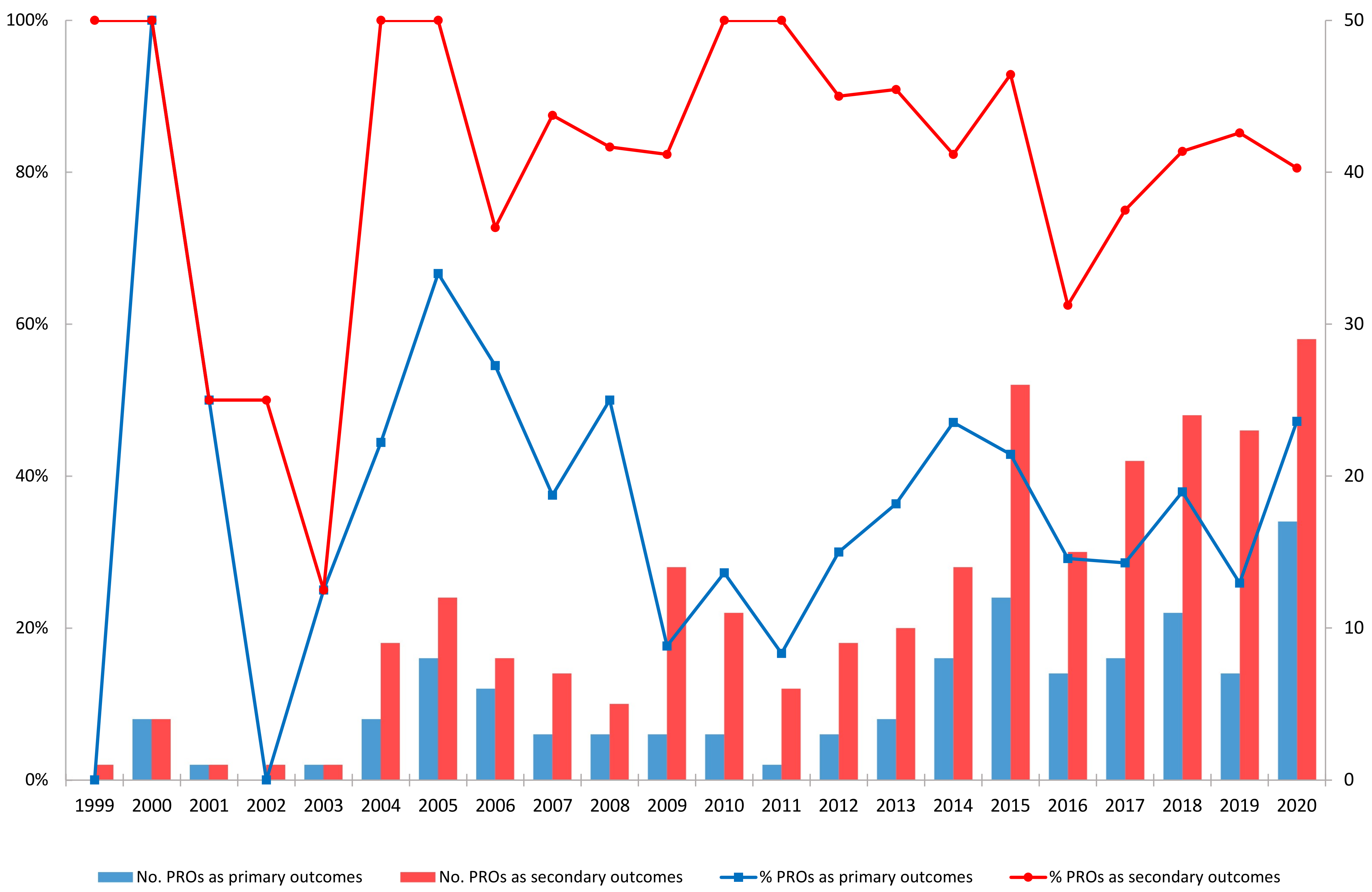
100%
80%
60%
40%
20%
0%
50
40
30
20
10
0
1999
2000
2001
2002
2003
2004
2005
2006
2007
2008
2009
2010
2011
2012
2013
2014
2015
2016
2017
2018
2019
2020
No. PROs as primary outcomes
No. PROs as secondary outcomes
% PROs as primary outcomes
% PROs as secondary outcomes

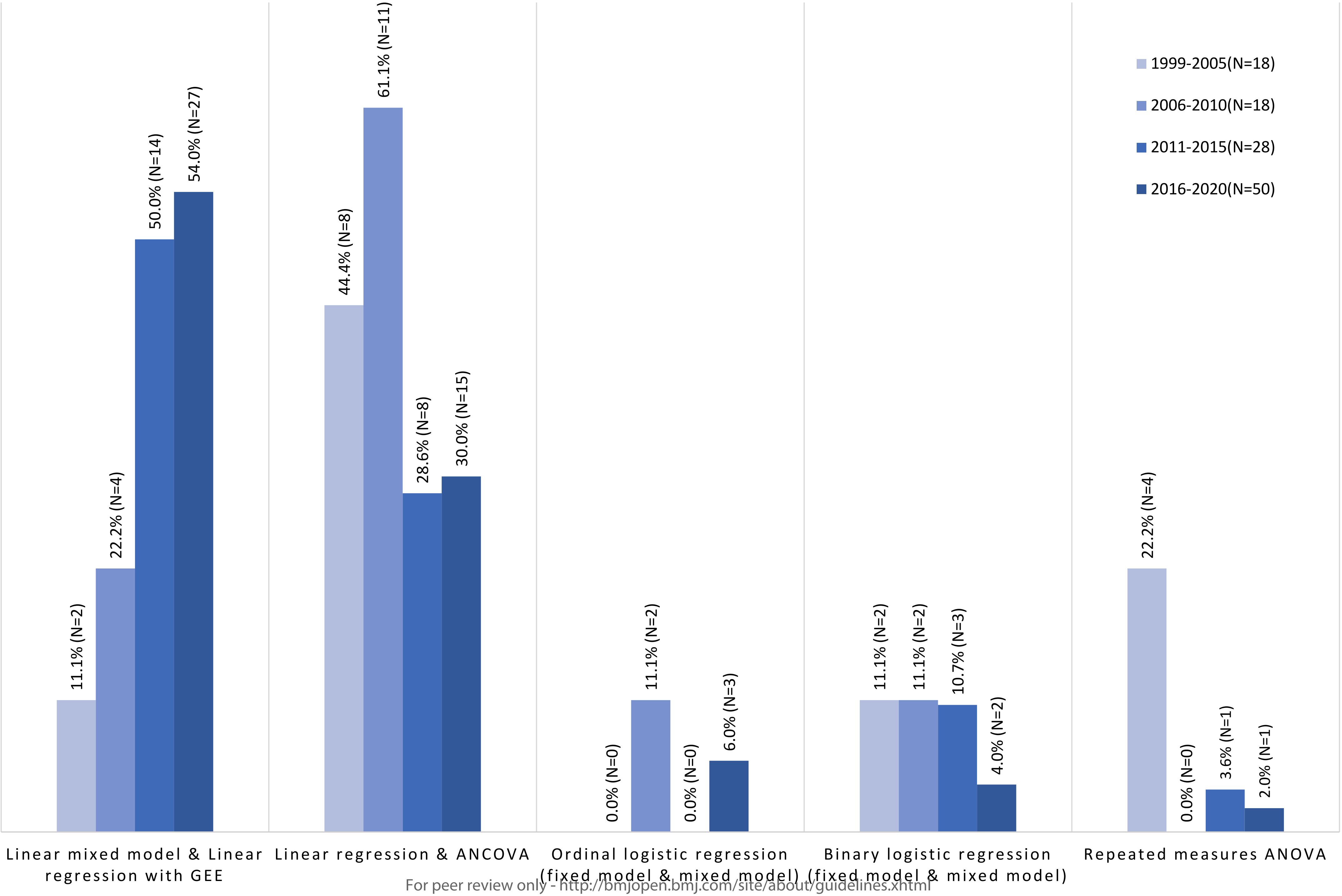

For peer review only - http://bmjopen.bmj.com/site/about/guidelines.xhtml

BMJ Open

A comprehensive review of statistical methods for analysing patient-reported outcomes (PROs) used as primary outcomes in randomised controlled trials (RCTs) published by the United Kingdom's Health Technology Assessment (HTA) Journal (1997-2020)

Journal:	*BMJ Open*
Manuscript ID	bmjopen-2021-051673.R1
Article Type:	Original research
Date Submitted by the Author:	31-Jul-2021

Complete List of Authors:	Qian, Yirui; The University of Sheffield School of Health and Related Research,<br>Walters, Stephen; The University of Sheffield School of Health and Related Research<br>Jacques, Richard; The University of Sheffield School of Health and Related Research<br>Flight, Laura ; The University of Sheffield School of Health and Related Research

**Primary Subject Heading**:	Public health
Secondary Subject Heading:	Health economics, Public health, Research methods
Keywords:	STATISTICS & RESEARCH METHODS, AUDIT, PUBLIC HEALTH

SCHOLARONE™ Manuscripts

I, the Submitting Author has the right to grant and does grant on behalf of all authors of the Work (as defined in the below author licence), an exclusive licence and/or a non-exclusive licence for contributions from authors who are: i) UK Crown employees; ii) where BMJ has agreed a CC-BY licence shall apply, and/or iii) in accordance with the terms applicable for US Federal Government officers or employees acting as part of their official duties; on a worldwide, perpetual, irrevocable, royalty-free basis to BMJ Publishing Group Ltd ("BMJ") its licensees and where the relevant Journal is co-owned by BMJ to the co-owners of the Journal, to publish the Work in this journal and any other BMJ products and to exploit all rights, as set out in our licence.

The Submitting Author accepts and understands that any supply made under these terms is made by BMJ to the Submitting Author unless you are acting as an employee on behalf of your employer or a postgraduate student of an affiliated institution which is paying any applicable article publishing charge ("APC") for Open Access articles. Where the Submitting Author wishes to make the Work available on an Open Access basis (and intends to pay the relevant APC), the terms of reuse of such Open Access shall be governed by a Creative Commons licence – details of these licences and which Creative Commons licence will apply to this Work are set out in our licence referred to above.

Other than as permitted in any relevant BMJ Author's Self Archiving Policies, I confirm this Work has not been accepted for publication elsewhere, is not being considered for publication elsewhere and does not duplicate material already published. I confirm all authors consent to publication of this Work and authorise the granting of this licence.

For peer review only

A comprehensive review of statistical methods for analysing patient-reported outcomes (PROs) used as primary outcomes in randomised controlled trials (RCTs) published by the United Kingdom's Health Technology Assessment (HTA) Journal (1997-2020)

Yirui Qian[1], Stephen J Walters[1], Richard M Jacques[1], Laura Flight[1]

[1]School of Health and Related Research, University of Sheffield, Sheffield, UK

Corresponding author:

Professor Stephen Walters

Email: s.j.walters@sheffield.ac.uk

School of Health and Related Research
University of Sheffield
30 Regent St
Sheffield
S1 4DA

Word count: 4600 (excluding title page, abstract, references, figures and tables)

Keywords: STATISTICS & RESEARCH METHODS, AUDIT, PUBLIC HEALTH.

For peer review only

Abstract

Objectives: To identify how frequently patient-reported outcomes (PROs) are used as primary and/or secondary outcomes in RCTs and to summarise what statistical methods are used for the analysis of PROs.

Design: Comprehensive review.

Setting: RCTs funded and published by the United Kingdom's (UK) National Institute for Health Research (NIHR) Health Technology Assessment (HTA) Programme.

Data sources and eligibility: HTA reports of RCTs published between January 1997 and December 2020 were reviewed.

Data extraction: Information relating to PROs use and analysis methods was extracted.

Primary and secondary measures: The frequency of using PROs as primary and/or secondary outcomes; statistical methods that were used for the analysis of PROs as primary outcomes.

Results: In this review, 37.6% (114/303) of trials used PROs as primary outcomes, and 82.8% (251/303) of trials used PROs as secondary outcomes from 303 NIHR HTA reports of RCTs. In the 114 RCTs where the PRO was the primary outcome, the most used PRO was Short-Form 36 (8/114), the most popular methods for multivariable analysis were linear mixed model (45/114), linear regression (29/114) and analysis of covariance (ANCOVA) (13/114); logistic regression was applied for binary and ordinal outcomes in 14/114 trials; and the repeated measures analysis was used in 39/114 trials.

Conclusion: The majority of trials used PROs as primary and/or secondary outcomes. Conventional methods such as linear regression are widely used, despite the potential violation of their assumptions. In recent years, there is an increasing trend of using complex models (e.g. with mixed effects). Statistical methods developed to address these violations when analysing PROs, such as beta-binomial regression, are not routinely used in practice. Future research will focus on evaluating available statistical methods for the analysis of PROs.

For peer review only

Article Summary

Strengths and limitations of this study

- Patient-reported outcomes (PROs) are increasingly used as clinical outcomes, and the use of appropriate statistical methods will ensure reliable estimates are produced to support decision making.
- This review summarises the up-to-date statistical methods that have been used for the primary analysis of PROs in 114 randomised controlled trials (RCTs) published by the United Kingdom's National Institute for Health Research (NIHR) Health Technology Assessment (HTA) Journal between January 1997 and December 2020, and it is the largest comprehensive review of trials on the use of PROs and the corresponding statistical methods for the analysis of PROs to date.
- Most studies apply conventional statistical methods (e.g. linear regression) for the analysis of PROs despite the potential violation of the model assumptions; and even though specialist statistical methods have been developed to solve this problem, these methods are rarely used in practice.
- There was a lack of explicit reporting of PROs in some trials. Although assumptions have been made based on the context where some required information was not explicitly stated, it is possible the extracted data was inconsistent with researchers' intention.
- This study only included trials published in the NIHR HTA Journal as the information relating to PROs in other journals are not reported in as much detail and the HTA Journal intends to publish all NIHR funded studies, regardless of their findings.

For peer review only

INTRODUCTION

Patient-reported outcomes (PROs) are health or health-related outcomes of health interventions reported by patients themselves. PROs enable health researchers to measure, analyse and compare clinical outcomes from the patient perspective and provide clinical effectiveness outcomes to support decision making. The descriptive and scoring system of a PRO can transform subjective descriptions of health to numerical scores in a range of dimensions;[1] and this transformation quantifies and allows the statistical analysis of these health-related outcomes.

Randomised controlled trials (RCTs) are regarded as the gold standard for evaluating the effectiveness of interventions.[2,3] The randomisation process in a well-conducted RCT can reduce the selection bias and allocation bias, and inform the causality of the treatment on responses.[4] These traits of RCTs can simplify the analysis of PRO data. But the RCT methodology does not bypass the possible systematic error from sources such as invalid measurements (e.g. poorly or mistakenly filled PRO forms, ceiling or floor effects of the PRO scales), publication bias or selective reporting of statistical analyses.

Despite these traits of RCTs, it can still be complex to analyse PRO data in RCT settings for the following reasons.

First, one PRO can generate multiple outcomes and can be reported in different forms – using different score types such as subscales or summary scores, generating quality-adjusted survivals (i.e. the integration of the quality-of-life function over the observed period of survival, such as quality-adjusted life years), producing dichotomised outcomes (e.g. proportion of responders with a PRO score above or below a particular pre-specified value or cut-off), and producing ordinal outcomes using several cut-offs. The various forms can result in multiple endpoints, and potentially increase the complexity of analysing PRO data.

Second, PRO data is likely to be discrete, skewed, and bounded (i.e. with ceiling effect and floor effects).[5] When analysing PRO data using a general linear model (including t-test, ANOVA, ANCOVA and linear regression), there are a number of assumptions[6]:

1. The values of the outcome variable should have a Normal distribution for each value of the explanatory variable. This assumption means that the residuals are Normally distributed and should have a mean of zero;
2. Constant variance or homoscedasticity of the outcome variable at each value of the explanatory variable;
3. The relationship between the outcome variable and the explanatory variable should be linear;

For peer review only

4. Independent observations in the sample.

These assumptions such as Normality of residuals and linear relationship between outcome independent variable and explanatory dependent variables are likely to be violated.[7,8]

Also, the application of statistical methods might vary according to PRO data and the aim of statistical analysis, but these features (multidimensional, discrete, skewed, and bounded) of PRO data may obscure the decision on what statistical methods need to be applied for the data analysis.

An inappropriate statistical analysis of PROs can result in unreliable estimands of clinical effectiveness and accordingly fail to provide accurate and robust results for decision-making, with wider confidence intervals or larger errors. [9,10] For example, patients may fail to receive effective treatment because this treatment is falsely shown not to be clinically effective based on inaccurate estimands; vice-versa, patients may receive treatment which may potentially harm their health when unreliable evidence supports the use of this treatment. Thus, applying appropriate statistical methods for the analysis of PROs is crucial to reduce biases of estimands, to accurately evaluate clinical effectiveness, and to support the healthcare decision-making.

This study aims to firstly identify how frequently PROs have been used as primary or secondary clinical outcomes in reports of RCTs published in the United Kingdom's (UK) National Institute for Health Research (NIHR) Health Technology Assessment (HTA) Journal; and secondly, when the PRO was the primary outcome for trial, to summarise the statistical methods used to analyse the outcome.

METHODS

Definition of a Patient-Reported Outcome (PRO)

There are several definitions of a PRO. For the purpose of this review, a patient-reported outcome measure (PROM) was defined as a questionnaire that measures health or a health-related outcome as a result of health interventions reported by patients themselves without any interpretation by clinicians or any other proxies. A patient-reported outcome (PRO) is an umbrella term for outcomes used to measure patients' perceptions of health-related quality of life (HRQoL), broadly quality of life (QoL), health status, satisfaction with the treatment and health conditions.[1,11–13]

Trial Identification

Reports of RCTs published in the UK's NIHR HTA Journal between 1 January 1997 and 31 December 2020 that defined and reported a PRO as clinical endpoints or outcomes for the trial

For peer review only

were systematically identified and reviewed. The HTA Journal was chosen because, in comparison to major medical journals, the information related to the trial and the PROs are reported in more detail. Information related to the use of PROs included the frequency of using PROs as clinical outcomes; whether the PROs were used as primary and/or secondary outcomes; the characteristics of the PROs when they were used as primary outcomes; and when the PRO was the primary outcome, the statistical methods used for the analysis of the PRO data. The identification of HTA reports of RCTs used the same search strategy as previous work.[14] The selection of trials with PROs was conducted by one reviewer (YQ). Three reviewers (SW, RJ, and LF) conducted quality assurance checks on 30% of the included papers after the data extraction was completed, and disagreements were discussed to achieve consensus.

Inclusion and exclusion criteria

The studies included in this review satisfied the following criteria: 1) individually randomised controlled trials; 2) trials with at least one PRO as the primary outcome; and 3) trials with the statistical analysis conducted for the PRO. Studies excluded from this review included cluster RCTs as these have specific statistical issues; influenza trials as these rarely use PROs as clinical outcomes; adaptive or group sequential trials as these have different statistical issues that may influence the choice of analysis; follow-on studies and pilot studies.

PROs identified in this review can be well-established measures from previous studies with feasibility, reliability and validity tested, or self-developed measures by researchers alongside trials. For studies with measures which were not clearly defined as a PRO in the trial, various methods were taken to identify whether the measure was categorised as a PRO, including retrieving the cited paper that developed the measure, identifying signal words such as ‘carers’ and ‘physicians’ for rating or assessing patients’ outcomes in the measure description, and referring to other papers that developed or applied the outcome measure. According to our definition of PROs, trials that only recruited patients, or trials that recruited both patients and proxies when patients were unable to complete PROs were included; trials that only used proxies as informants to complete the PROs were excluded in order to avoid the cases where clinicians respond to health outcome measures on the patients’ behalf.

Trials using the product of PROs, such as a dichotomised outcome and quality-adjusted survivals, were included. Trials that used PROs only as primary cost-effectiveness outcomes, but not as clinical primary outcomes were excluded. Even if the statistical methods were proposed, trials that did not actually conduct the statistical analysis were excluded.

Data Extraction

The following information was extracted from the included trials:

For peer review only

1. Characteristics of the trials with PROs as primary outcomes, including the number of participants randomised and analysed, the baseline and post-randomisation assessment, the most frequently used PROs, and special types of PROs (including patient satisfaction, preference-based and proxy-reported).
2. Statistical methods conducted for the primary analysis of the PROs, including the study population, the specific statistical methods, the adjustment for baseline score or other covariates, involvement of random effects, robust standard errors and bootstrapping techniques, repeated measures analysis, and strategies for missing data.
3. The quality of reporting PROs, including whether there is a clear definition or justification of the primary outcomes or primary endpoints, statistical methods, and covariates.

For the purpose of this review, the statistical methods were broadly categorised into two categories: 'univariable methods' that do not adjust for any other covariates except the randomised group (e.g. *t*-test, Chi-squared test, and simple linear regression) and 'multivariable methods' that have one or more explanatory variables (e.g. baseline score) in addition to the randomised group (e.g. multiple linear regression). The multivariable methods were further classified according to the categories of generalized linear models (GzLM), including linear regression, analysis of covariance (ANCOVA), binary logistic regression, ordinal logistic regression, and their extensions for correlated responses such as models with coefficients estimated by generalised estimating equations (GEEs) and mixed effect models. Repeated measures analysis for PROs with more than one post-baseline assessments were classified into four categories: response feature analysis (i.e. using summary measures, such as the area under the curve or post-randomisation mean score), generalized linear mixed models (GzLMM), GzLM with parameters estimated by GEE, and repeated measures analysis of variance (ANOVA).[5,15]

Patient and Public Involvement

Patients and the public were not involved in any way in this study.

RESULTS

In total, 1356 reports were published by the HTA Journal between 1 January 1997 and 31 December 2020, and 940 reports were excluded after screening the titles and abstracts. In the remaining 416 reports, 115 were excluded for various reasons (Figure 1). In the 303 published individually randomised controlled trials, 37.6% (114/303) of trials used PROs as primary outcomes and 82.8% (251/303) of trials used PROs as secondary outcomes. Two trials with

For peer review only

PROs as primary outcomes were excluded as they were closed without conducting a statistical analysis of the data using the statistical methods that were proposed in the report.[16,17] It should be noted that the first RCT with a PRO as a clinical outcome was published in the HTA Journal in 1999,[18] and the earlier reports published in the HTA Journal were mainly systematic reviews.

All included trials that used PROs as primary outcomes also used PROs as secondary outcomes. The trend of using PROs as clinical outcomes in trials between 1999 to 2020 is shown in Figure 2. Except for the earlier years (1999-2003) with a small number of studies, the proportion of trials with PROs used as secondary outcomes (the red curve) is approximately two times higher than the proportion of the trials with PROs as primary outcomes (the blue curve). Generally, there is an increase in using PROs as clinical outcomes in HTA trials.

Trial characteristics

In total, 83.1% (61,715/74,298) of the participants randomised in the 114 trials were used for the primary analysis (Table 1). The characteristics of these trials are summarised in Table 2. The most common design was a two-arm parallel group trial. More than a half trials were in either mental health (30/114) or musculoskeletal conditions (28/114). Most trials collected baseline assessments (101/114) and more than one post-baseline assessments (107/114). The maximum number of post-baseline assessment was 24 in a trial on eczema management for children.[19]

Table 1 Recruitment and retention of trial participants included from the 144 reports

Items	Mean	Median	SD	Min	Max	Total
Number of participants randomised	652	480	928	85	8,003	74,298
Number of participants analysed*	541	388	847	65	7,677	61,715

*number of participants analysed in the primary analysis of PROs; if multiple post-baseline assessments were used for primary outcome, the number of participants analysed at the longest primary endpoint was taken. SD, standard deviation.

Most trials (107/114) clearly defined the primary outcomes; the sample size calculation implied the primary outcomes for the trial was a PRO in six trials that did not explicitly specify the primary outcome, and one trial defined PROs as main outcome measures but used an alternative outcome for the sample size calculation.[20] Table 3 summarises the PROs used as primary outcomes in four or more included trials. The most popular PROs were mainly generic (i.e. SF-36/SF-6D and EQ-5D) and depression-specific (i.e. BDI, HADS and PHQ). Eight trials used more than one PRO as the primary outcomes, and fourteen trials used non-PRO clinical outcomes as co-primary outcomes.

For peer review only

Table 2 Trial design and assessments characteristics of the 114 trials included in the review

Items		No	%	Total
Trial design				114
	Parallel group	102	87.2	
	Factorial	3	2.6	
	Crossover	0	0.0	
	Other*	9	7.7	
Number of arms				114
	2	83	70.9	
	3	21	17.9	
	4	5	4.3	
	>4	5	4.3	
Clinical area				114
	Mental Health	30	25.6	
	Musculoskeletal	28	23.9	
	Obstetrics and gynaecology	9	7.7	
	Gastrointestinal	7	6.0	
	Respiratory	5	4.3	
	Stroke	5	4.3	
	Primary Care	4	3.4	
	Cardiovascular	4	3.4	
	Cancer/Oncology	3	2.6	
	Dermatology	4	3.4	
	Other^	15	12.8	
Number of trials with a baseline assessment of the PRO				114
		101	86.3	
Timing of primary outcome post-baseline assessments				114
	<1 month	7	6.0	
	1-6 months	28	23.9	
	6-18 months	50	42.7	
	>18 months	27	23.1	
	Missing$	2	1.7	
Number of post-baseline assessments				114
	1	5	4.3	
	2	41	35.0	
	3	29	24.8	
	4	19	16.2	
	>4	18	15.4	
	Missing$	2	1.7	

*patient preference/Zelen's.

^chronic fatigue, minor surgery, multiple sclerosis, neurosurgery, paediatric, sleep disorders, urology, vascular.

$Two trials did not specify the timing and number of post-baseline assessments.

Preference-based PROs were used in six trials as primary outcomes, including five[21–25] that used the EQ-5D and one[26] that used the SF-6D. Seven trials used the quality-adjusted survivals, including three [22,23,25] that used EQ-5D and four[27–30] that used specific PROs for estimation. Patient satisfaction was used as primary outcome in two trials.[31,32] Proxies were recruited in six trials[19,33–37] that primarily aimed to collect patient-reported outcomes, and only recruited proxies for patients who were unable to complete the PROs. Seven included trials used self-developed PROs as primary or coprimary outcome. Most of these PROs had one item based on Likert scale or visual analogue scale (VAS), except for one trial[38] that specially developed a REFLUX questionnaire with 31 items to generate the QoL of patients with gastro-oesophageal reflux disease. Health outcomes assessed by investigators were not included as primary outcomes, e.g. four trials[39–41] using Quality of Life Scale or Rankin Scale that were completely assessed by investigators were excluded.

Different score types were used to summarise the PRO primary outcomes. Most trials (98/114) used one score type to report the primary outcome, and the rest trials used two types of scores. Summary scores were the most popular way to measure PROs (83/114), followed by subscales (30/114) and single items (16/114), including Likert scale (11/114) and VAS (5/114).

Table 3 The most frequently used PROs as primary outcomes in the included trials

Patient-Reported Outcomes	Abbr.	No	%	Ref
Short Form-36	SF-36	8	7.0	[42–45]
Short Form 6-Dimension	SF-6D			
Beck Depression Inventory	BDI	7	6.1	[46]
Hospital Anxiety and Depression Scale	HADS	5	4.4	[47]
EuroQol-5 Dimension	EQ-5D	5	4.4	[48–50]
Patient Health Questionnaire	PHQ	5	4.4	[51]
Oxford Shoulder Score	OSS	4	3.5	[52]
Other*		90	78.9	
Total^		124	108.8	

*Only PROs that were used in four or more trials are listed separately.

^The total number of included trials is 114. Eight trials used more than one PRO as primary outcomes, including two trials that used three PROs, and six trials that used two PROs as primary outcomes.

Statistical methods for the primary analysis of PROs

The majority of trials stated the proportion of missing PRO data (109/114), developed strategies to deal with missing data (99/114), imputed missing data using various methods such as mean imputation and last observation carried forward (89/114). In 40/114 studies, missing data were imputed as part of a sensitivity analysis to check the robustness of the primary analysis strategy which did not consider missing data.

Intention-to-treat (ITT) analysis (i.e. analysis based on the treatment assignment of all participants but not the actual treatment received)[53], including ITT with and without missing data imputation, was used in 111/114 trials, and 46 trials used other study populations such as per protocol analysis (i.e. analysis based on the patients who completed the originally treatment assigned), as treated, or complier average causal effect analysis (i.e. analysis of the treatment effect based on the subgroup that completed the originally treatment assigned) for the secondary or sensitivity analysis.

The statistical methods applied for primary analyses were clearly defined in 79/114 trials, and the use of univariable or multivariable methods for primary analyses were justified in 92/114 trials. Except for two trials that did not specify the timing and number of post-randomisation assessments,[54,55] 72/114 clearly defined the single timepoint used for the primary analysis (e.g. x-months post-baseline), and 40/114 used the repeated post-baseline outcomes for the primary analysis.

The statistical methods used for the primary analysis of PROs are shown in Table 4. The linear mixed model (45/114) and linear regression (29/114) and ANCOVA (13/114) were the most popular methods. Among the 45 trials using linear mixed models for the primary analysis, 23 trials conducted a repeated measures analysis, and the rest did not consider the repeated post-baseline outcomes in the primary analysis. Three trials used GzLM with coefficients estimated by GEE for the longitudinal primary analysis, including one as an extension to ordinal logistic regression[56] and two as an extension to linear regression.[39,57] Only one trial with quality-adjusted survivals used Cox regression for the primary analysis.[25]

Bootstrapped confidence intervals were calculated after using a general linear model (including *t*-test, ANCOVA, linear regression) or linear mixed model in six trials[18,20,22,37,58,59] due to the skewness of PRO scores, and one additional trial did not conduct any other statistical analysis except calculating the bootstrapped confidence interval for the treatment estimate[60]. Robust standard errors can be used to estimate confidence intervals and the calculation of test statistics (and associated P-values). Six trials[37,56,61–63] reported using robust standard errors based on regression methods for the primary analysis, and three trials[57,64,65] reported using robust standard errors for the longitudinal analysis in the non-primary analysis.

For peer review only

There were 106 trials that used multivariable methods, of which (98/106) clearly reported the covariates adjusted in the primary analysis. Among them, 85 trials adjusted for baseline score of the PRO, and three trials[66–68] modelled the change of PRO from baseline in the primary analysis. The use of random effects for the primary analysis of PROs were clearly specified in 47 trials: 44 used linear mixed models, and the rest three used repeated measures ANOVA [69], binary logistic mixed model[27], and ordinal logistic mixed model[32] respectively. The most common random factors applied in the multivariable methods were therapists, centres (i.e. hospital sites), and individual patients.

Trend of using statistical methods for the primary analysis of PROs

Figure 3 shows the trend of using different statistical methods over the observed period. There is an increasing trend for using more complex models (mixed models and GLzM with GEE) in most recent years for the analysis of PROs. The linear regression, ANCOVA and repeated measures ANOVA were the most popular regression methods used from 1999 to 2010, used in around 63% of trials averagely. But this popularity dropped to 28.6% during 2011-2015 and 30.0% during 2016-2020. In contrast, the proportion of trials using the linear mixed model and linear regression with GEE for correlated outcomes increased across the observation period, from 11.1% to 54.0%. While the use of binary logistic regression slightly decreased from 11.1% to 4.0% over time, the proportion of trials using ordinal logistic regression for the primary analysis remained small.

For peer review only

Table 4 Statistical methods for the main or primary analysis of PROs that are the primary outcomes of the trials

Statistical methods	N	%	Total
Univariable methods			27
t-test	11	40.7	
unadjusted regression methods*	7	25.9	
Chi-squared test	3	11.1	
Wilcoxon rank-sum test (Mann–Whitney U test)	4	14.8	
Kruskal-Wallis test	1	3.7	
log-rank test	1	3.7	
Multivariable methods^			106
linear mixed model	45	42.5	
linear regression	28	27.4	
ANCOVA	13	12.3	
linear regression with GEE	2	1.9	
binary logistic regression	8	7.5	
binary logistic mixed model	1	0.9	
ordinal logistic regression	4	3.8	
ordinal logistic mixed model	1	0.9	
repeated measures ANOVA	6	5.7	
survival analysis	1	0.9	
Repeated measures analysis			39
linear mixed model	23	59.0	
response feature analysis$	7	17.9	
repeated measures ANOVA	6	15.4	
GzLM with GEE	3	7.7	

*In seven trials using unadjusted regression methods with no other covariates in the model besides randomised group, three trials used linear regression, one used linear mixed model, two used ordinal logistic regression and one used binary logistic regression.

^106 trials used multivariable methods for the analysis of PROs, including four trials that used two different methods for the primary analysis of PROs.

$In seven trials using response feature analysis (with quality-adjusted survivals) in primary analysis, four used linear regression or ANCOVA, two used linear mixed models, and one used survival analysis.

ANCOVA, analysis of covariance; ANOVA, analysis of variance; GzLM, generalised linear model; GEE, generalised estimating equation.

DISCUSSION

This study has systematically conducted a review which summarised how frequently PROs have been used and what statistical methods have been applied for the primary analysis of PROs in RCTs published by the UK's NIHR HTA Journal between 1997 and 2020. This review found that 82.8% (251/303) used PROs as primary or secondary outcome, and 37.6% (114/303) used PROs as primary outcomes. Though conventional methods (such as the *t*-test and multiple linear regression & ANCOVA) have been widely used, there is a growing trend of using more complex methods such as linear mixed models (with both fixed and random effects) for the analysis of PROs over the observed period.

The general linear model (including *t*-test, ANOVA, ANCOVA and linear regression) and linear mixed model were widely used over the past two decades, which could possibly result from the frequent use of continuous data type of response variables. The trend of using complex models could result from the increasingly complexity of trial designs and from recommendations on using linear mixed models over repeated measures ANOVA for the longitudinal analysis of PROs.[5,70] There is a decrease in the use of binary logistic regression as the dichotomised outcome gathers less information from the PROs compared to other score types.[71]

There is a gap between statistical methods developed by methodologists and statistical methods used for the analysis of PROs in trials. For example, Arostegui, Núñez-Antón and Quintana recommend the use of ordinal logistic regression with random effects model, beta-binomial regression or beta-logit-normal regression for continuous or ordinal PRO data after testing distributional assumptions.[70,72] However, only one of the five included trials that used ordinal logistic regression for the primary analysis considered random effects.[32] Neither beta-binomial regression nor beta-logit-normal regression were used by the 114 trials. Interestingly, the international standards for the analysis of PROs in cancer trials by the SISAQOL Consortium neither recommended the use of ordinal regression nor compared ordinal regression with other statistical methods.[73] The conventional methods are popular since the estimands produced by simple models can remain robust regardless of the violation of model assumptions.[7] In addition, the difficulty in applying newly developed models in statistical software and the complexity to explain estimands of these models may make researchers more reluctant to use them.

PRO outcomes tend to generate data with discrete, skewed, and bounded distributions that are not Normally distributed, and the assumptions for statistical methods such as the t-test, linear regression and ANCOVA may not be valid. However, Heeren and D'Agostino[74] have demonstrated the robustness of the two independent samples t-test when applied to three-, four-

For peer review only

and five-point ordinal scaled data using assigned scores, in sample sizes as small as 20 subjects per group. Sullivan and D'Agostino[75] have expanded this work to account for a covariate when the outcome is ordinal in nature. They again assign numeric scores to the distinct response categories and compare means between treatment groups adjusting for a covariate reflecting a baseline assessment measured on the same scale. Their simulation study shows that in the presence of three-, four- and five-point ordinal data and small sample sizes (as low as 20 per group) that both ANCOVA and the two independent sample t-test on difference scores are robust and produce actual significance levels close to the nominal significance levels.

Furthermore, statistical theory says that if the distribution of an outcome variable is Normal, so will be the distribution of the sample mean for that outcome variable. Much more importantly, even if the distribution of the outcome is not Normal, that of the sample mean will become closer to the Normal distribution as the sample size gets larger. This is a consequence of the Central Limit Theorem (CLT). The Normal distribution is strictly only the limiting form of the sampling distribution as the sample size increases to infinity, but it provides a remarkably good approximation to the sampling distribution even when the sample size is small, and the distribution of the outcome variable is far from Normal.[76] Thus conventional statistical methods such as the t-test, linear regression/ANCOVA for analysing PROs are robust to the violation of assumptions for moderate to large sample sizes.[77]

To the best of our knowledge this study is by far the largest review of trials (with 114 studies) published by the HTA journal which analysed the frequency of using PROs and the statistical methods for the analysis of PROs. The reviews by Pe et al of trials in breast cancer; Hamel et al (lung cancer), and Fiteni et al (lung cancer) had sample sizes of 66, 33, 27 articles respectively.[9,10,78] Compared to other reviews that concentrate on only one clinical area (i.e. oncology),[9,10,78–81] this review summarised details in the frequency of using PROs and applying statistical methods in RCTs with a range of clinical areas.

It is noteworthy that the proportions of trials using PROs reported in this review represents the average rate of HTA trials focusing on different clinical areas, and when considering specific disease(s) or selecting different database(s), the proportions may vary. For example, Pe et al[10] identified 3/66 (5%) and 46/66 (70%) randomised controlled trials of locally advanced and metastatic breast cancer using PROs as primary and secondary endpoints respectively. Marandino et al[82] reviewed 446 cancer trials published in major journals between 2012 and 2016, and found that PRO or QoL was a primary end point in five trials (1.1%), a secondary end point in 195 trials (43.7%), an exploratory endpoint in 36 trials (8.1%), while in the remaining 210 (47.1%) QoL was not listed at all among study end points. Our review found that three of eighteen cancer trials (17%) used PROs as primary outcomes, and 13/18 (72%)

For peer review only

used PROs as secondary outcomes. Our results showed that PROs were more frequently used for health problems such as mental and musculoskeletal disease.

This study has the following limitations. First, this review only looked at publicly funded trials in the UK, which may represent a limitation in terms of the generalisability of the findings. It is possible that statistical methods are used differently in industry funded trials or in trials in other countries. However, as the NIHR HTA Journal intends to publish all NIHR funded projects, it has less publication bias compared to journals that only publish positive outcomes, and the information related to PROs in other journals are not reported in as much detail. The extracted statistical methods for the analysis of PROs from this review are consistent with those included from other similar reviews.[9,10,78–81] Second, there might be other appropriate methods for the analysis of PROs that were not included in this review. This review mainly analysed RCTs with PROs as primary outcomes because primary outcomes and the corresponding statistical methods were more explicitly reported.

Third, trials with PROs only used as cost-effectiveness outcomes were excluded. This is because the statistical strategies for clinical effectiveness and cost-effectiveness outcomes may vary, and cost-effectiveness analysis (CEA) produces both cost and clinical effectiveness outcomes. If PROs for CEA were included in this review, the proportion of included trials would increase, as there were some studies using EQ-5D for the primary CEA. It could be argued that the analysis of effectiveness estimated by PROs in CEA also requires appropriate statistical methods, but estimands calculated for a health economic analysis could be different from those for a clinical analysis as they hold different purposes to conduct these analyses. Therefore, we believe it is justified to make this exclusion.

We used a broad definition for a PRO and a small number of trials (seven) used PROs that were specifically developed for the trial and were not validated in another external study. The inclusion of such non-validated instruments as primary outcomes should be discouraged, and may have affected the results, although the characteristics of these PROs (Likert or VAS) are similar to those of the PROs that have been formally validated. We believe that it is not unreasonable to assume that the statistical analysis of such outcomes would be similar to the analysis of validated PROs.

Another potential limitation is the large time window, 1997-2020, chosen for the review. This may introduce some variability and potential heterogeneity in the trials included in the review, but on the positive side it allows to test time trends in the type of statistical methods used in the trials.

For peer review only

Last, the information related to PROs and statistical methods were not clearly reported in some trials. Although assumptions have been made based on the context where some required information was not explicitly stated, it is possible that the data extracted was inconsistent with researchers' intention. However, as the data have been extracted for all reports by one reviewer there is consistency in the interpretation and assumptions made.

To produce explicit reports, it is recommended that researchers follow specific guidelines that can instruct the reporting of using PROs in RCT papers and protocols such as the CONSORT PRO Extension,[13] SPIRIT PRO Extension,[83] and the standards for the analysis of PROs in cancer RCTs.[73] In addition, a clear classification of the terminology of the statistical methods is desired. It is a historical problem that the names of statistical methods are confusing (e.g. general linear model vs. generalized linear model), and multiple terms can be used to describe the same method, for example, the proportional odds model, ordered logit model and ordinal logistic regression refer to the same regression technique. Thus, researchers should be clear and cautious when describing the exact statistical method for the analysis.

In addition to the abovementioned issues, other obstacles are also worth attention when analysing PROs in RCT settings, which can be broadly classified into three domains: first, the statistics domain – inadequate understanding of basic statistical concepts,[84] incorrect procedures used to carry out statistical analysis and incorrect statistical inference (e.g. secretly hypothesizing after results are known);[85,86] second, the PROs domain – application of invalid measurements,[87] lack of comparability among the results produced by various PROs[88]; and the third domain, reporting and publishing – publication bias and selective reporting of trials and their associated outcomes.[88,89]

In conclusion, the majority of trials funded by the NIHR HTA Programme used PROs as primary and/or secondary outcomes. Although there is an increasing trend of using complex models (e.g. mixed effects), conventional methods such as linear regression remain widely used for the analysis of PROs, despite the potential violation of their assumptions. Statistical methods developed to address these violations when analysing PROs, such as beta-binomial regression, are not routinely used in practice. Various methods for the analysis of PROs have been identified from this review, but it is still unknown which methods are the most appropriate for the analysis of PRO data. Future research will focus on evaluating available statistical methods and make recommendations on using different methods for the analysis of PRO data.

For peer review only

Figure Legends

Figure 1 Flow diagram for the inclusion and exclusion of trials published in the Health Technology Assessment Journal from 1997 to 2020

Figure 2 Number and proportion of trials using PROs as primary and/or secondary outcomes from 1999 to 2020

Figure 3 The percentage of trials using multivariable methods for the primary analysis of PROs from 1999 to 2020

(N = ?) denotes the number of trials published in the specified period. As the survival analysis was only used in one trial, it is not shown in this graph. As this graph only summarised multivariable methods and one trial could use two or more multivariable methods for the primary analysis, the number of trials summarised in this graph may not equal the total number of included trials.

For peer review only

Contributors All authors contributed to the study concept and study design. YQ and RJ contributed to the selection of data. YQ conducted the data extraction and data analysis. SW, RJ, and LF contributed to the quality assurance check of the data. YQ drafted the manuscript. All authors critically revised the manuscript and approved the final manuscript.

Competing interests The PhD study of Qian is financially sponsored by the University of Sheffield and China Scholarship Council (Grant No. 201908890049). Prof. Walters, Dr. Jacques, and Dr. Flight received funding across various projects by NIHR. Prof. Walters is a National Institute for Health Research (NIHR) Senior Investigator (NF-SI-0617-10012) supported by the NIHR for this research project. The views expressed in this publication are those of the author(s) and not necessarily those of the NIHR, NHS or the UK Department of Health and Social Care. These organisations had no role in the study design; in the collection, analysis, and interpretation of the data; in the writing of the report; or in the decision to submit the paper for publication.

Funding Qian is sponsored jointly by the University of Sheffield and China Scholarship Council (Grant No. 201908890049). Prof. Walters, Dr. Jacques, and Dr. Flight received funding across various projects by NIHR. Prof. Walters is a National Institute for Health Research (NIHR) Senior Investigator (NF-SI-0617-10012) supported by the NIHR for this research project. The views expressed in this publication are those of the author(s) and not necessarily those of the NIHR, NHS or the UK Department of Health and Social Care. These organisations had no role in the study design; in the collection, analysis, and interpretation of the data; in the writing of the report; or in the decision to submit the paper for publication.

Patient consent for publication This review does not involve recruiting new participants or analysing individual participants data. Individual informed consent was obtained to take part in the original trials by primary investigators.

Data availability statement The information extracted in this review is based on published trials in the Health Technology Assessment Journal. The data extracted from the HTA Journal supporting the finding of this study is available upon reasonable request from Yirui Qian at yqian21@sheffield.ac.uk.

Ethical statement: The information extracted in this review is based on published HTA trials where ethics approvals were obtained by the original trial teams. This review does not involve recruiting new participants or analysing individual participants, and the original participants cannot be identified from this review.

For peer review only

REFERENCES

1 Brazier J, Ratcliffe J, Salomon JA, *et al.* Introduction to the measurement and valuation of health. In: *Measuring and Valuing Health Benefits for Economic Evaluation (2 ed.)*. 2016. 7–30. doi:10.1093/med/9780198725923.001.0001

2 Akobeng AK. Understanding randomised controlled trials. Arch. Dis. Child. 2005;**90**:840–4. doi:10.1136/adc.2004.058222

3 Altman DG. Better reporting of randomised controlled trials: The CONSORT statement. BMJ. 1996;**313**:570–1. doi:10.1136/bmj.313.7057.570

4 Moher D, Hopewell S, Schulz KF, *et al.* CONSORT 2010 explanation and elaboration: updated guidelines for reporting parallel group randomised trials. *BMJ* 2010;**340**. doi:10.1136/bmj.c869

5 Walters SJ. *Quality of Life Outcomes in Clinical Trials and Health-Care Evaluation*. Chichester, UK: : John Wiley & Sons, Ltd 2009. doi:10.1002/9780470840481

6 Walters S, Campbell J, Machin D. *Medical statistics : a textbook for the health sciences*. Wiley-Blackwell 2021.

7 Walters SJ, Campbell MJ. The use of bootstrap methods for analysing health-related quality of life outcomes (particularly the SF-36). *Health Qual Life Outcomes* 2004;**2**. doi:10.1186/1477-7525-2-70

8 Lumley T, Diehr P, Emerson S, *et al.* The importance of the normality assumption in large public health data sets. Annu. Rev. Public Health. 2002;**23**:151–69. doi:10.1146/annurev.publhealth.23.100901.140546

9 Hamel JF, Saulnier P, Pe M, *et al.* A systematic review of the quality of statistical methods employed for analysing quality of life data in cancer randomised controlled trials. Eur. J. Cancer. 2017;**83**:166–76. doi:10.1016/j.ejca.2017.06.025

10 Pe M, Dorme L, Coens C, *et al.* Statistical analysis of patient-reported outcome data in randomised controlled trials of locally advanced and metastatic breast cancer: a systematic review. *Lancet Oncol* 2018;**19**:e459–69. doi:10.1016/S1470-2045(18)30418-2

11 Fitzpatrick R, Davey C, Buxton MJ, *et al.* Evaluating patient-based outcome measures for use in clinical trials. *Health Technol Assess (Rockv)* 1998;**2**. doi:10.3310/hta2140

12 Mokkink LB, Terwee CB, Patrick DL, *et al.* The COSMIN checklist for assessing the methodological quality of studies on measurement properties of health status

For peer review only

measurement instruments: An international Delphi study. *Qual Life Res* 2010;**19**:539–49. doi:10.1007/s11136-010-9606-8

13 Calvert M, Blazeby J, Altman DG, *et al.* Reporting of patient-reported outcomes in randomized trials: The CONSORT PRO extension. J. Am. Med. Assoc. 2013;**309**:814–22. doi:10.1001/jama.2013.879

14 Walters SJ, Dos Anjos Henriques-Cadby IB, Bortolami O, *et al.* Recruitment and retention of participants in randomised controlled trials: A review of trials funded and published by the United Kingdom Health Technology Assessment Programme. *BMJ Open* 2017;**7**:15276. doi:10.1136/bmjopen-2016-015276

15 Schober P, Vetter TR. Repeated measures designs and analysis of longitudinal data: If at first you do not succeed-try, try again. *Anesth Analg* 2018;**127**:569–75. doi:10.1213/ANE.0000000000003511

16 Williams NH, Jenkins A, Goulden N, *et al.* Subcutaneous injection of Adalimumab Trial compared with Control (SCIATiC): A randomised controlled trial of adalimumab injection compared with placebo for patients receiving physiotherapy treatment for sciatica. *Health Technol Assess (Rockv)* 2017;**21**:1–179. doi:10.3310/hta21600

17 Mihaylov S, Stark C, McColl E, *et al.* Stepped treatment of older adults on laxatives. The STOOL trial. *Health Technol Assess (Rockv)* 2008;**12**. doi:10.3310/hta12130

18 Morrell CJ, Spiby H, Stewart P, *et al.* Costs and benefits of community postnatal support workers: A randomised controlled trial. *Health Technol Assess (Rockv)* 2000;**4**. doi:10.3310/hta4060

19 Santer M, Rumsby K, Ridd MJ, *et al.* Adding emollient bath additives to standard eczema management for children with eczema: The BATHE RCT. *Health Technol Assess (Rockv)* 2018;**22**:1–116. doi:10.3310/hta22570

20 Kerry S, Hilton S, Patel S, *et al.* Routine referral for radiography of patients presenting with low back pain: Is patients' outcome influenced by GPs' referral for plain radiography? *Health Technol Assess (Rockv)* 2000;**4**. doi:10.3310/hta4200

21 Gazzard G, Konstantakopoulou E, Garway-Heath D, *et al.* Selective laser trabeculoplasty versus drops for newly diagnosed ocular hypertension and glaucoma: The LiGHT RCT. *Health Technol Assess (Rockv)* 2019;**23**:1–101. doi:10.3310/hta23310

22 Sharples L, Everett C, Singh J, *et al.* Amaze: A double-blind, multicentre randomised

For peer review only

controlled trial to investigate the clinical effectiveness and cost-effectiveness of adding an ablation device-based maze procedure as an adjunct to routine cardiac surgery for patients with pre-existing atrial fibrillation. *Health Technol Assess (Rockv)* 2018;**22**. doi:10.3310/hta22190

23 Watson AJM, Cook J, Hudson J, *et al.* A pragmatic multicentre randomised controlled trial comparing stapled haemorrhoidopexy with traditional excisional surgery for haemorrhoidal disease: The eTHoS study. *Health Technol Assess (Rockv)* 2017;**21**:1–223. doi:10.3310/hta21700

24 Brittenden J, Cotton SC, Elders A, *et al.* Clinical effectiveness and cost-effectiveness of foam sclerotherapy, endovenous laser ablation and surgery for varicose veins: Results from the comparison of LAser, Surgery and foam Sclerotherapy (CLASS) randomised controlled trial. *Health Technol Assess (Rockv)* 2015;**19**:1–341. doi:10.3310/hta19270

25 Russell I, Edwards R, Gliddon A, *et al.* Cancer of oesophagus or gastricus: New assessment of technology of endosonography (COGNATE): Report of pragmatic randomised trial. *Health Technol Assess (Rockv)* 2013;**17**:1–13. doi:10.3310/hta17390

26 Michaels JA, Campbell WB, Brazier JE, *et al.* Randomised clinical trial, observational study and assessment of cost-effectiveness of the treatment of varicose veins (REACTIV trial). *Health Technol Assess (Rockv)* 2006;**10**:1–114. doi:10.3310/hta10130

27 Pickard R, Goulao B, Carnell S, *et al.* Open urethroplasty versus endoscopic urethrotomy for recurrent urethral stricture in men: The open rct. *Health Technol Assess (Rockv)* 2020;**24**:1–110. doi:10.3310/hta24610

28 Hewison J, Nixon J, Fountain J, *et al.* Amniocentesis results: Investigation of anxiety. The ARIA trial. *Health Technol Assess (Rockv)* 2006;**10**. doi:10.3310/hta10500

29 Bedson E, Bell D, Carr D, *et al.* Folate augmentation of treatment - Evaluation for depression (folated): Randomised trial and economic evaluation. *Health Technol Assess (Rockv)* 2014;**18**:1–159. doi:10.3310/hta18480

30 Williams JG, Alam MF, Alrubaiy L, *et al.* Comparison Of iNfliximab and ciclosporin in STeroid Resistant Ulcerative Colitis: Pragmatic randomised trial and economic evaluation (CONSTRUCT). *Health Technol Assess (Rockv)* 2016;**20**:1–322. doi:10.3310/hta20440

31 Townsend J, Wolke D, Hayes J, *et al.* Routine examination of the newborn: The EMREN study. Evaluation of an extension of the midwife role including a randomised

For peer review only

controlled trial of appropriately trained midwives and paediatric senior house officers. *Health Technol Assess (Rockv)* 2004;**8**. doi:10.3310/hta8140

32 Cooper K, Breeman S, Scott NW, *et al.* Laparoscopic supracervical hysterectomy compared with second-generation endometrial ablation for heavy menstrual bleeding: The HEALTH RCT. *Health Technol Assess (Rockv)* 2019;**23**. doi:10.3310/hta23530

33 Dennis M, Forbes J, Graham C, *et al.* Fluoxetine to improve functional outcomes in patients after acute stroke: The focus rct. *Health Technol Assess (Rockv)* 2020;**24**:1–94. doi:10.3310/hta24220

34 Dennis M, Lewis S, Cranswick G, *et al.* FOOD: A multicentre randomized trial evaluating feeding policies in patients admitted to hospital with a recent stroke. *Health Technol Assess (Rockv)* 2006;**10**:1–91. doi:10.3310/hta10020

35 Banerjee S, Hellier J, Romeo R, *et al.* Study of the use of antidepressants for depression in dementia: The HTA-SADD trial- A multicentre, randomised, double-blind, placebo-controlled trial of the clinical effectiveness and cost-effectiveness of sertraline and mirtazapine. *Health Technol Assess (Rockv)* 2013;**17**:1–43. doi:10.3310/hta17070

36 Francis NA, Ridd MJ, Thomas-Jones E, *et al.* A randomised placebo-controlled trial of oral and topical antibiotics for children with clinically infected eczema in the community: The ChildRen with eczema, antibiotic management (CREAM) study. *Health Technol Assess (Rockv)* 2016;**20**. doi:10.3310/hta20190

37 Weindling AM, Cunningham CC, Glenn SM, *et al.* Additional therapy for young children with spastic cerebral palsy: A randomised controlled trial. *Health Technol Assess (Rockv)* 2007;**11**. doi:10.3310/hta11160

38 Grant A, Wileman S, Ramsay C, *et al.* The effectiveness and cost-effectiveness of minimal access surgery amongst people with gastro-oesophageal reflux disease – a UK collaborative study. The reflux trial. *Health Technol Assess (Rockv)* 2008;**12**:1–218. doi:10.1136/bmj.a2664

39 Lewis SW, Davies L, Jones PB, *et al.* Randomised controlled trials of conventional antipsychotic versus new atypical drugs, and new atypical drugs versus clozapine, in people with schizophrenia responding poorly to, or intolerant of, current drug treatment. *Health Technol Assess (Rockv)* 2006;**10**:1–94. doi:10.3310/hta10170

40 Langhorne P, Wu O, Rodgers H, *et al.* A very early rehabilitation trial after stroke (AVERT): a Phase III, multicentre, randomised controlled trial. *Health Technol Assess (Rockv)* 2017;**21**:1–119. doi:10.3310/hta21540

For peer review only

41 Bath PM, Woodhouse LJ, Appleton JP, *et al.* Triple versus guideline antiplatelet therapy to prevent recurrence after acute ischaemic stroke or transient ischaemic attack: The TARDIS RCT. *Health Technol Assess (Rockv)* 2018;**22**:1–75. doi:10.3310/hta22480

42 J E Ware Jr, C D Sherbourne. The MOS 36-item short-form health survey (SF-36). I. Conceptual framework and item selection - PubMed. *Med Care* 1992;**30**:473–83.https://pubmed.ncbi.nlm.nih.gov/1593914/ (accessed 8 Mar 2021).

43 McHorney CA, Ware JE, Raczek AE. The MOS 36-item short-form health survey (Sf-36): II. Psychometric and clinical tests of validity in measuring physical and mental health constructs. *Med Care* 1993;**31**:247–63. doi:10.1097/00005650-199303000-00006

44 McHorney CA, Ware JE, Rachel Lu JF, *et al.* The MOS 36-item short-form health survey (SF-36): III. Tests of data quality, scaling assumptions, and reliability across diverse patient groups. *Med Care* 1994;**32**:40–66. doi:10.1097/00005650-199401000-00004

45 Brazier J, Roberts J, Deverill M. The estimation of a preference-based measure of health from the SF-36. *J Health Econ* 2002;**21**:271–92. doi:10.1016/S0167-6296(01)00130-8

46 Beck AT, Steer RA, Carbin MG. Psychometric properties of the Beck Depression Inventory: Twenty-five years of evaluation. *Clin Psychol Rev* 1988;**8**:77–100. doi:10.1016/0272-7358(88)90050-5

47 Zigmond AS, Snaith RP. The Hospital Anxiety and Depression Scale. *Acta Psychiatr Scand* 1983;**67**:361–70. doi:10.1111/j.1600-0447.1983.tb09716.x

48 The EuroQol Group. EuroQol - a new facility for the measurement of health-related quality of life. *Health Policy (New York)* 1990;**16**:199–208. doi:10.1016/0168-8510(90)90421-9

49 Dolan P. Modeling Valuations for EuroQol Health States. *Med Care* 1997;**35**:1095–108. doi:10.1097/00005650-199711000-00002

50 Herdman M, Gudex C, Lloyd A, *et al.* Development and preliminary testing of the new five-level version of EQ-5D (EQ-5D-5L). *Qual Life Res* 2011;**20**:1727–36. doi:10.1007/s11136-011-9903-x

51 Spitzer RL, Kroenke K, Williams JBW. Validation and utility of a self-report version of PRIME-MD: The PHQ Primary Care Study. *J Am Med Assoc* 1999;**282**:1737–44. doi:10.1001/jama.282.18.1737

For peer review only

52 Dawson J, Fitzpatrick R, Carr A. Questionnaire on the perceptions of patients about shoulder surgery. *J Bone Jt Surg - Ser B* 1996;**78**:593–600. doi:10.1302/0301-620x.78b4.0780593

53 Montedori A, Bonacini MI, Casazza G, *et al.* Modified versus standard intention-to-treat reporting: Are there differences in methodological quality, sponsorship, and findings in randomized trials? A cross-sectional study. *Trials* 2011;**12**:58. doi:10.1186/1745-6215-12-58

54 Little P, Hobbs FDR, Moore M, *et al.* PRImary care Streptococcal Management (PRISM) study: In vitro study, diagnostic cohorts and a pragmatic adaptive randomised controlled trial with nested qualitative study and cost-effectiveness study. *Health Technol Assess (Rockv)* 2014;**18**:1–101. doi:10.3310/hta18060

55 Little P, Turner S, Rumsby K, *et al.* Dipsticks and diagnostic algorithms in urinary tract infection: Development and validation, randomised trial, economic analysis, observational cohort and qualitative study. *Health Technol Assess (Rockv)* 2009;**13**. doi:10.3310/hta13190

56 Kennedy TM, Chalder T, McCrone P, *et al.* Cognitive behavioural therapy in addition to antispasmodic therapy for irritable bowel syndrome in primary care: Randomised controlled trial. Health Technol. Assess. (Rockv). 2006;**10**. doi:10.3310/hta10190

57 Molassiotis A, Russell W, Hughes J, *et al.* The effectiveness and cost-effectiveness of acupressure for the control and management of chemotherapy-related acute and delayed nausea: Assessment of Nausea in Chemotherapy Research (ANCHoR), a randomised controlled trial. *Health Technol Assess (Rockv)* 2013;**17**:1–114. doi:10.3310/hta17260

58 Shawo L, Bhattaraio N, Cant R, *et al.* An extended stroke rehabilitation service for people who have had a stroke: The extras rct. *Health Technol Assess (Rockv)* 2020;**24**:1–202. doi:10.3310/hta24240

59 Symmons D, Tricker K, Roberts C, *et al.* Aggressive versus symptomatic therapy in established rheumatoid arthritis. *Health Technol Assess (Rockv)* 2005;**9**.

60 Wiggins M, Oakley A, Roberts I, *et al.* The social support and family health study: A randomised controlled trial and economic evaluation of two alternative forms of postnatal support for mothers living in disadvantaged inner-city areas. *Health Technol Assess (Rockv)* 2004;**8**. doi:10.3310/hta8320

61 Kennedy ADM, Sculpher MJ, Coulter A, *et al.* A multicentre randomised controlled trial assessing the costs and benefits of using structured information and analysis of

For peer review only

women's preferences in the management of menorrhagia. *Health Technol Assess (Rockv)* 2003;**7**. doi:10.3310/hta7080

62 Francis NA, Gillespie D, White P, *et al.* C-reactive protein point-of-care testing for safely reducing antibiotics for acute exacerbations of chronic obstructive pulmonary disease: The PACE RCT. *Health Technol Assess (Rockv)* 2020;**24**. doi:10.3310/hta24150

63 Beard DJ, Davies LJ, Cook JA, *et al.* Total versus partial knee replacement in patients with medial compartment knee osteoarthritis: The topkat rct. *Health Technol Assess (Rockv)* 2020;**24**. doi:10.3310/hta24200

64 Chalder M, Wiles NJ, Campbell J, *et al.* A pragmatic randomised controlled trial to evaluate the cost-effectiveness of a physical activity intervention as a treatment for depression: The treating depression with physical activity (TREAD) trial. *Health Technol Assess (Rockv)* 2012;**16**. doi:10.3310/hta16100

65 Goodyer IM, Reynolds S, Barrett B, *et al.* Cognitive-behavioural therapy and short-term psychoanalytic psychotherapy versus brief psychosocial intervention in adolescents with unipolar major depression (IMPACT): A multicentre, pragmatic, observer-blind, randomised controlled trial. *Health Technol Assess (Rockv)* 2017;**21**:1–93. doi:10.3310/hta21120

66 Goodacre S, Cohen J, Bradburn M, *et al.* The 3Mg trial: a randomised controlled trial of intravenous or nebulised magnesium sulphate versus placebo in adults with acute severe asthma. *Health Technol Assess (Rockv)* 2014;**18**. doi:10.3310/hta18220

67 Williams MA, Williamson EM, Heine PJ, *et al.* Strengthening and stretching for rheumatoid arthritis of the hand (SARAH). A randomised controlled trial and economic evaluation. *Health Technol Assess (Rockv)* 2015;**19**:221. doi:10.3310/hta19190

68 Clarke CE, Patel S, Ives N, *et al.* Clinical effectiveness and cost-effectiveness of physiotherapy and occupational therapy versus no therapy in mild to moderate Parkinson's disease: a large pragmatic randomised controlled trial (PD REHAB). *Health Technol Assess* 2016;**20**:1–96. doi:10.3310/hta20630

69 Peveler R, Kendrick T, Buxton M, *et al.* A randomised controlled trial to compare the cost-effectiveness of tricyclic antidepressants, selective serotonin reuptake inhibitors and lofepramine. *Health Technol Assess* 2005;**9**. doi:10.3310/hta9160

70 Arostegui I, Núñez-Antón V, Quintana JM. Statistical approaches to analyse patient-reported outcomes as response variables: An application to health-related quality of life.

For peer review only

Stat Methods Med Res 2012;**21**:189–214. doi:10.1177/0962280210379079

71 Shields A, Coon C, Hao Y, *et al.* Patient-reported outcomes for US oncology labeling: Review and discussion of score interpretation and analysis methods. *Expert Rev Pharmacoeconomics Outcomes Res* 2015;**15**:951–9. doi:10.1586/14737167.2015.1115348

72 Arostegui I, Núñez-Antón V, Quintana JM. Analysis of the short form-36 (SF-36): The beta-binomial distribution approach. *Stat Med* 2007;**26**:1318–42. doi:10.1002/sim.2612

73 Coens C, Pe M, Dueck AC, *et al.* International standards for the analysis of quality-of-life and patient-reported outcome endpoints in cancer randomised controlled trials: recommendations of the SISAQOL Consortium. Lancet Oncol. 2020;**21**:e83–96. doi:10.1016/S1470-2045(19)30790-9

74 Heeren T, D'Agostino R. Robustness of the two independent samples t-test when applied to ordinal scaled data. *Stat Med* 1987;**6**:79–90. doi:10.1002/sim.4780060110

75 Sullivan LM, D'Agostino RB. Robustness and power of analysis of covariance applied to ordinal scaled data as arising in randomized controlled trials. *Stat Med* 2003;**22**:1317–34. doi:10.1002/sim.1433

76 Armitage P, Berry G, Matthews JNS. *Statistical methods in medical research.* 4th editio. Oxford, Blackwell Science 2002.

77 Walters SJ. *Quality of Life Outcomes in Clinical Trials and Health-Care Evaluation*. Chichester: Wiley 2009. doi:10.1002/9780470840481

78 Fiteni F, Anota A, Westeel V, *et al.* Methodology of health-related quality of life analysis in phase III advanced non-small-cell lung cancer clinical trials: A critical review. *BMC Cancer* 2016;**16**. doi:10.1186/s12885-016-2152-1

79 Fiteni F, Ray I Le, Ousmen A, *et al.* Health-related quality of life as an endpoint in oncology phase i trials: A systematic review. *BMC Cancer* 2019;**19**. doi:10.1186/s12885-019-5579-3

80 Nielsen LK, Abildgaard N, Jarden M, *et al.* Methodological aspects of health-related quality of life measurement and analysis in patients with multiple myeloma. Br. J. Haematol. 2019;**185**:11–24. doi:10.1111/bjh.15759

81 Turner-Bowker DM, Hao Y, Foley C, *et al.* The use of patient-reported outcomes in advanced breast cancer clinical trials: a review of the published literature. Curr. Med. Res. Opin. 2016;**32**:1709–17. doi:10.1080/03007995.2016.1205005

For peer review only

82 Marandino L, La Salvia A, Sonetto C, *et al.* Deficiencies in health-related quality-of-life assessment and reporting: a systematic review of oncology randomized phase III trials published between 2012 and 2016. Published Online First: 2018. doi:10.1093/annonc/mdy449

83 Calvert M, Kyte D, Mercieca-Bebber R, *et al.* Guidelines for inclusion of patient-reported outcomes in clinical trial protocols the spirit-pro extension. *J Am Med Assoc* 2018;**319**:483–94. doi:10.1001/jama.2017.21903

84 Gigerenzer G, Gaissmaier W, Kurz-Milcke E, *et al.* Helping doctors and patients make sense of health statistics. *Psychol Sci Public Interes Suppl* 2007;**8**:53–96. doi:10.1111/j.1539-6053.2008.00033.x

85 Hollenbeck JR, Wright PM. Harking, Sharking, and Tharking: Making the Case for Post Hoc Analysis of Scientific Data. J. Manage. 2017;**43**:5–18. doi:10.1177/0149206316679487

86 Russell Localio A, Stack CB, Meibohm AR, *et al.* Inappropriate statistical analysis and reporting in medical research: Perverse incentives and institutional solutions. Ann. Intern. Med. 2018;**169**:577–8. doi:10.7326/M18-2516

87 Chang EM, Gillespie EF, Shaverdian N. Truthfulness in patient-reported outcomes: factors affecting patients' responses and impact on data quality. *Patient Relat Outcome Meas* 2019;**Volume 10**:171–86. doi:10.2147/prom.s178344

88 Yordanov Y, Dechartres A, Atal I, *et al.* Avoidable waste of research related to outcome planning and reporting in clinical trials. *BMC Med* 2018;**16**. doi:10.1186/s12916-018-1083-x

89 Ioannidis JPA, Greenland S, Hlatky MA, *et al.* Increasing value and reducing waste in research design, conduct, and analysis. Lancet. 2014;**383**:166–75. doi:10.1016/S0140-6736(13)62227-8

For peer review only

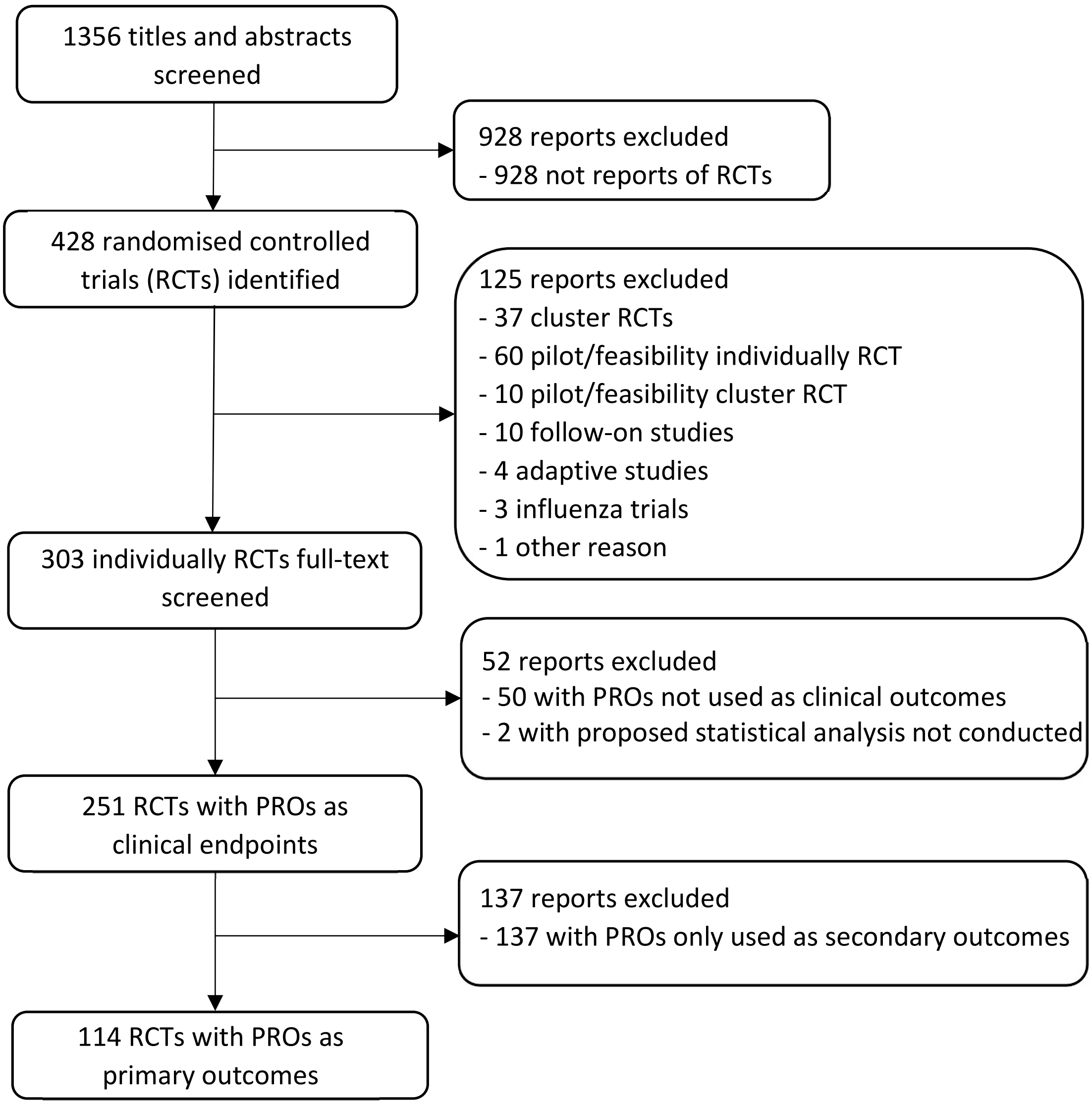
1356 titles and abstracts screened
928 reports excluded
- 928 not reports of RCTs
428 randomised controlled trials (RCTs) identified
125 reports excluded
- 37 cluster RCTs
- 60 pilot/feasibility individually RCT
- 10 pilot/feasibility cluster RCT
- 10 follow-on studies
- 4 adaptive studies
- 3 influenza trials
- 1 other reason
303 individually RCTs full-text screened
52 reports excluded
- 50 with PROs not used as clinical outcomes
- 2 with proposed statistical analysis not conducted
251 RCTs with PROs as clinical endpoints
137 reports excluded
- 137 with PROs only used as secondary outcomes
114 RCTs with PROs as primary outcomes

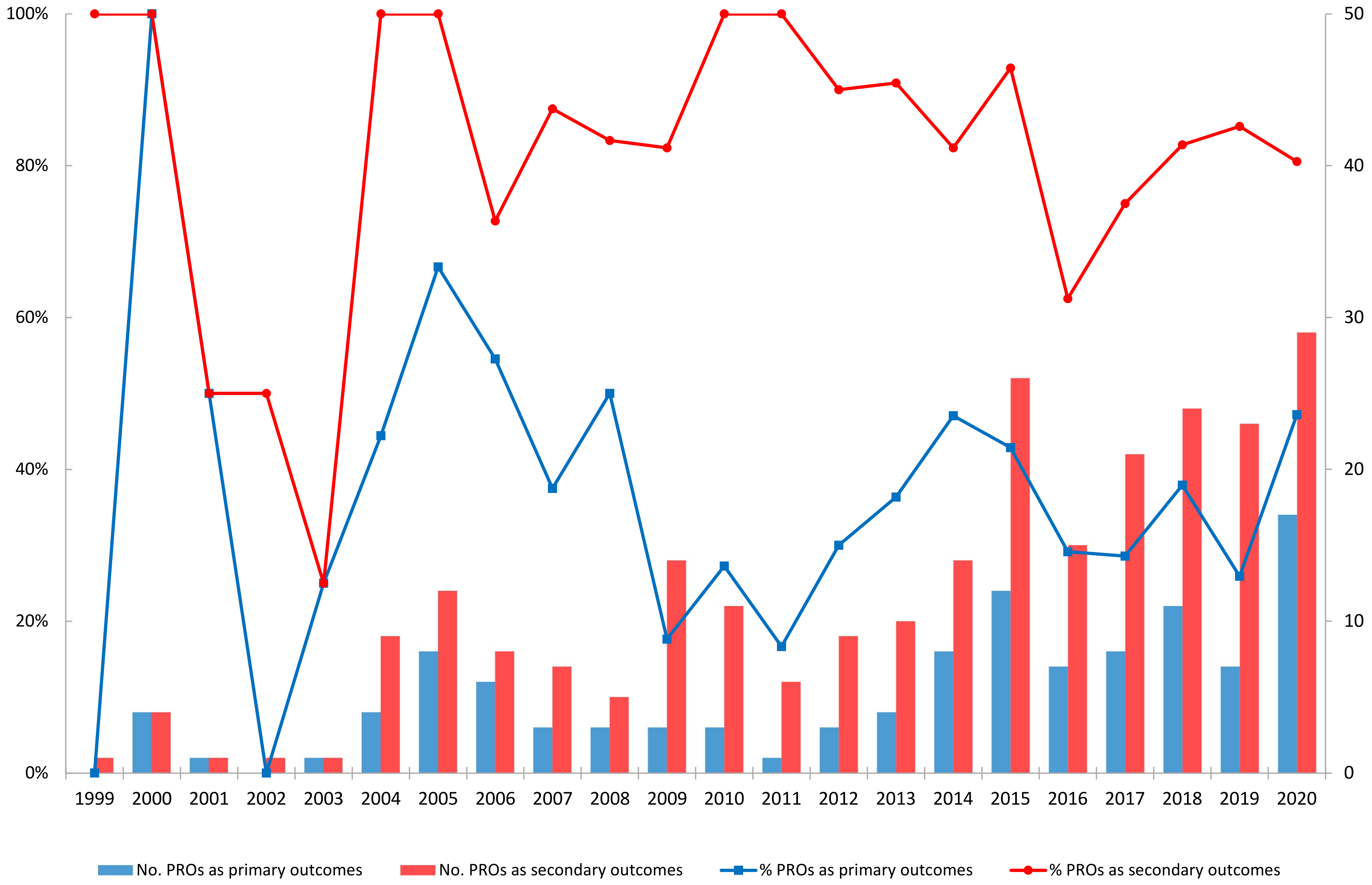
100%
80%
60%
40%
20%
0%
50
40
30
20
10
0
1999
2000
2001
2002
2003
2004
2005
2006
2007
2008
2009
2010
2011
2012
2013
2014
2015
2016
2017
2018
2019
2020
No. PROs as primary outcomes
No. PROs as secondary outcomes
% PROs as primary outcomes
% PROs as secondary outcomes

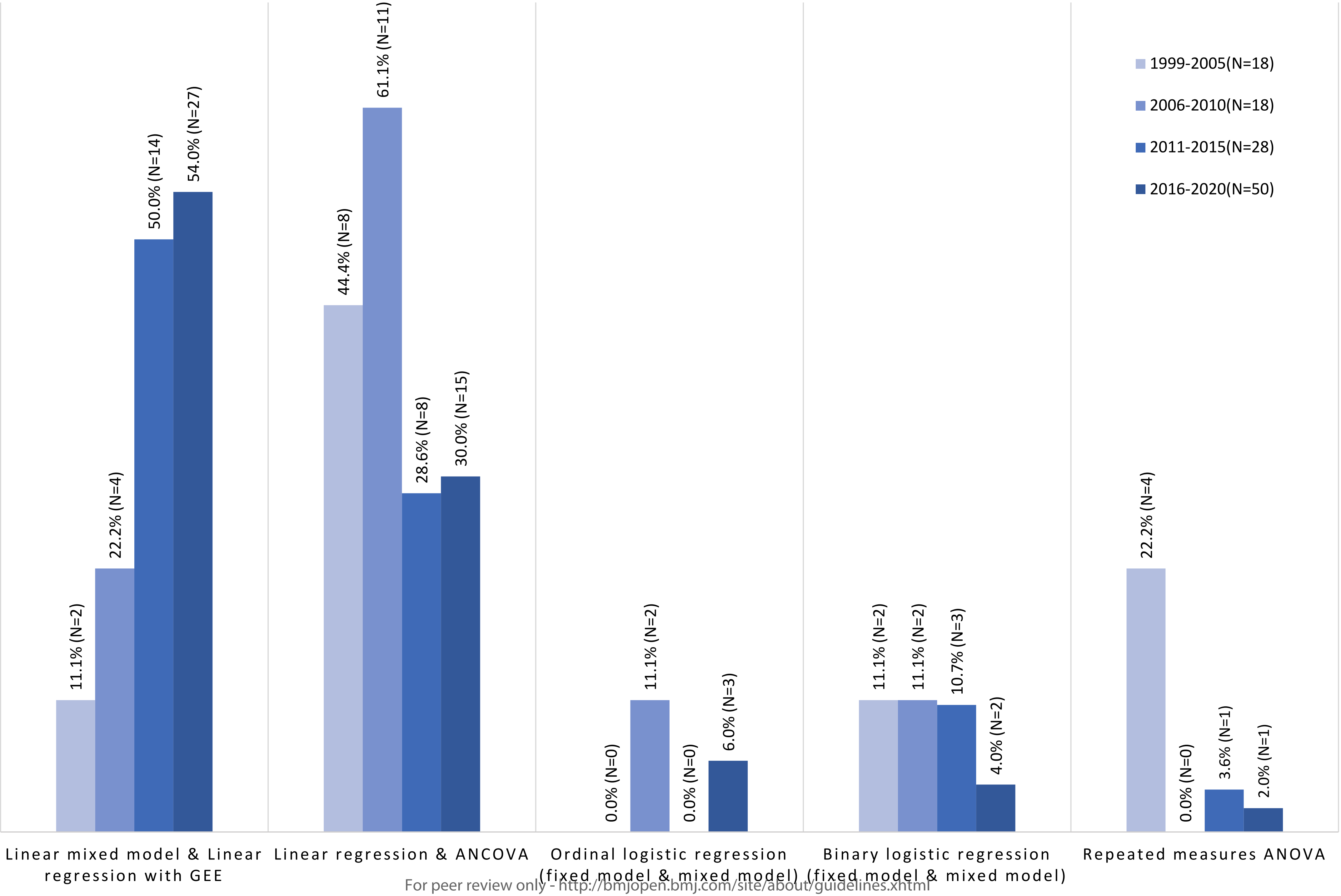
1999-2005(N=18)
2006-2010(N=18)
2011-2015(N=28)
2016-2020(N=50)
11.1% (N=2)
22.2% (N=4)
50.0% (N=14)
54.0% (N=27)
44.4% (N=8)
61.1% (N=11)
28.6% (N=8)
30.0% (N=15)
0.0% (N=0)
11.1% (N=2)
0.0% (N=0)
6.0% (N=3)
11.1% (N=2)
11.1% (N=2)
10.7% (N=3)
4.0% (N=2)
22.2% (N=4)
0.0% (N=0)
3.6% (N=1)
2.0% (N=1)
Linear mixed model & Linear regression with GEE
Linear regression & ANCOVA
Ordinal logistic regression (fixed model & mixed model)
Binary logistic regression (fixed model & mixed model)
Repeated measures ANOVA
